# Impact of model assumptions on demographic inferences: the case study of two sympatric mouse lemurs in northwestern Madagascar

**DOI:** 10.1186/s12862-021-01929-z

**Published:** 2021-11-02

**Authors:** Helena Teixeira, Jordi Salmona, Armando Arredondo, Beatriz Mourato, Sophie Manzi, Romule Rakotondravony, Olivier Mazet, Lounès Chikhi, Julia Metzger, Ute Radespiel

**Affiliations:** 1 Institute of Zoology, University of Veterinary Medicine Hannover, Foundation, Bünteweg 17, 30559 Hannover, Germany; 2 Laboratoire Évolution and Diversité Biologique (EDB UMR 5174), Université de Toulouse Midi-Pyrénées, CNRS, IRD, UPS, 118 Route de Narbonne, Bât. 4R1, 31062 Toulouse cedex 9, France; 3 Instituto Gulbenkian de Ciência, Rua da Quinta Grande, 6, 2780-156 Oeiras, Portugal; 4 Université de Toulouse, Institut National des Sciences Appliquées, Institut de Mathématiques de Toulouse, Toulouse, France; 5 Ecole Doctorale Ecosystèmes Naturels (EDEN), University of Mahajanga, 5 Rue Georges V - Immeuble KAKAL, Mahajanga Be, B.P. 652, 401 Mahajanga, Madagascar; 6 Faculté des Sciences, de Technologies et de l’Environnement, University of Mahajanga, 5 Rue Georges V - Immeuble KAKAL, Mahajanga Be, B.P. 652, 401 Mahajanga, Madagascar; 7 Institute of Animal Breeding and Genetics, University of Veterinary Medicine Hannover, Foundation, Bünteweg 17p, 30559 Hannover, Germany; 8 Veterinary Functional Genomics, Max Planck Institute for Molecular Genetics, Ihnestrasse 73, 14195 Berlin, Germany

**Keywords:** Quaternary climatic oscillations, Genomics, Demographic modelling, Madagascar, Mouse lemurs

## Abstract

**Background:**

Quaternary climate fluctuations have been acknowledged as major drivers of the geographical distribution of the extraordinary biodiversity observed in tropical biomes, including Madagascar. The main existing framework for Pleistocene Malagasy diversification assumes that forest cover was strongly shaped by warmer Interglacials (leading to forest expansion) and by cooler and arid glacials (leading to forest contraction), but predictions derived from this scenario for forest-dwelling animals have rarely been tested with genomic datasets.

**Results:**

We generated genomic data and applied three complementary demographic approaches (*Stairway Plot*, *PSMC* and *IICR*-simulations) to infer population size and connectivity changes for two forest-dependent primate species (*Microcebus murinus* and *M. ravelobensis*) in northwestern Madagascar. The analyses suggested major demographic changes in both species that could be interpreted in two ways, depending on underlying model assumptions (i.e., panmixia or population structure). Under panmixia, the two species exhibited larger population sizes across the Last Glacial Maximum (LGM) and towards the African Humid Period (AHP). This peak was followed by a population decline in *M. ravelobensis* until the present, while *M. murinus* may have experienced a second population expansion that was followed by a sharp decline starting 3000 years ago. In contrast, simulations under population structure suggested decreasing population connectivity between the Last Interglacial and the LGM for both species, but increased connectivity during the AHP exclusively for *M. murinus*.

**Conclusion:**

Our study shows that closely related species may differ in their responses to climatic events. Assuming that Pleistocene climatic conditions in the lowlands were similar to those in the Malagasy highlands, some demographic dynamics would be better explained by changes in population connectivity than in population size. However, changes in connectivity alone cannot be easily reconciled with a founder effect that was shown for *M. murinus* during its colonization of the northwestern Madagascar in the late Pleistocene. To decide between the two alternative models, more knowledge about historic forest dynamics in lowland habitats is necessary. Altogether, our study stresses that demographic inferences strongly depend on the underlying model assumptions. Final conclusions should therefore be based on a comparative evaluation of multiple approaches.

**Supplementary Information:**

The online version contains supplementary material available at 10.1186/s12862-021-01929-z.

## Background

Marked Quaternary climatic oscillations have been largely acknowledged as a major driver of evolutionary and biogeographical patterns of species worldwide [1, 2]. The present-day distribution, genetic diversity patterns and demography of many temperate and tropical species have been shaped by historical warming–cooling cycles that forced species to retract and expand according to their ecological requirements [2–8]. Accordingly, there is an increasing interest in reconstructing the climate, biome and fire regimes of the late Quaternary. Most studies focus on the well-pronounced climate fluctuations that occurred during the last Interglacial–Glacial cycle, which included the Last Interglacial (LIG; ca. 132–112 kyr; kyr = thousand years) and the Last Glacial Maximum (LGM; ca. 26.5–19 kyr) [9].

There is a general consensus that the climate during the LGM was globally cooler than today, even if the magnitude of the cooling was not spatially uniform across the globe [1, 10, 11]. However, there is less general knowledge about the effects of the last glaciation across the tropics [1, 11–13], partly due to the scarcity of high-resolution paleoenvironmental records in the southern tropics. In contrast to these uncertainties about the last Interglacial–Glacial cycle, the so-called African Humid Period (AHP; ca. 15 to 5 kyr [14–16], but timing differed slightly across Africa) represents a well-established climatic event in various African regions. The AHP was characterized by a sudden increase in summer precipitation that was followed by an abrupt shift toward more arid conditions, strongly impacting the vegetation cover across continental Africa and Madagascar [14, 17, 18]. The demographic history of a species, e.g., the timing and extent of population expansions or bottleneck events, should indirectly mirror past environmental fluctuations and can provide information about species resilience to past [19] and possibly future climatic oscillations.

Madagascar is a natural evolutionary laboratory, allowing to investigate how past climatic changes shaped species demographic history. First, the island is characterized by exceptional levels of species richness and endemism [20]. Second, Madagascar has been isolated from other landmasses for over 80 million years (Mya) [21] and exhibits marked environmental gradients. These conditions promoted multiple adaptive radiations and resulted in many cases of micro-endemism that evolved in response to a particular set of local or regional environmental and climatic conditions [22]. Third, Madagascar was one of the last major landmasses on Earth settled by humans (e.g., [23–27] but see [24, 28, 29]), which enables us to control for the confounding effect of the anthropogenic impact on endemic species demography until recent times. It has long been assumed that the climate in Madagascar was generally cooler and more arid during glaciations, and that the extent of the forest cover dramatically contracted during these periods [30–32], likely resulting in high levels of specialization [33]. However, solid evidence for historical forest cover dynamics across different regions and habitat types on the island is still missing.

**Fig. 1:**
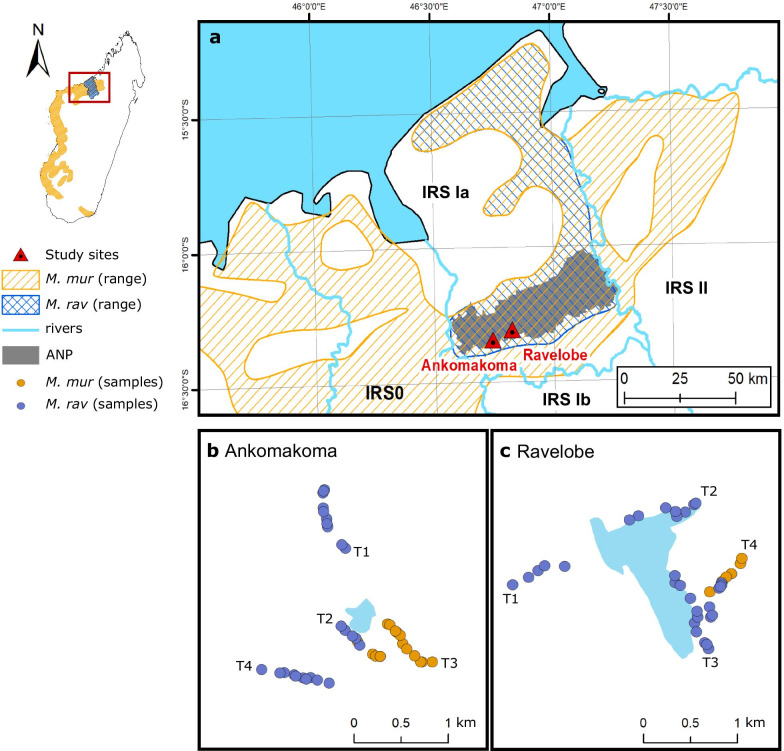
Study area and sampling strategy. **a** Distribution range of *M. murinus* and *M. ravelobensis* across northwestern Madagascar and location of study sites. Individual capture locations of mouse lemurs in **b** Ravelobe and **c** Ankomakoma along four forest transects that were arranged in proximity to one lake each (in blue). The two forest sites were approximately 10 km apart. **a** The river shape files were provided by [141] and the outlines of the Ankarafantsika National Park were obtained from the Protected Planet Database [142]. **b** and **c** Individual coordinates can be found in Additional file 1: Table S8. M.mur = *M. murinus*; M.rav = *M. ravelobensis*; ANP = Ankarafantsika National Park

More than 90% of the Malagasy species and almost all lemurs live exclusively in forests and woodlands [34]. Among the lemuriforms, mouse lemurs (*Microcebus spp.*) provide a suitable model for demographic studies, because they have a very young age at first reproduction (ca. 8 months) [35] and therefore a relatively short generation time (ca. 2.5 years) [36]. Moreover, as forest-dwelling species they should be susceptible to vegetation shifts, and their Pleistocene demographic dynamics can therefore be expected to correspond to past environmental changes. While mouse lemurs are typically microendemic species, *M. murinus* is the only mouse lemur species with a large geographic distribution, inhabiting various forest habitats from southern to northwestern Madagascar [37]. Across its range, *M. murinus* co-occurs with five other locally restricted mouse lemur species, including *M. ravelobensis* in one northern part of its distribution [38, 39]. *M. ravelobensis* occurs exclusively in the so-called Inter-River-System Ia (IRS Ia; Fig. 1a) delimited by the Betsiboka and Mahajamba rivers [40, 41]. The two species have most likely undergone very different evolutionary trajectories. While *M. ravelobensis* is thought to have diverged allopatrically from its sister species *M. bongolavensis* also distributed in northwestern Madagascar [40], *M. murinus* likely diverged allopatrically from its sister species *M. griseorufus* at about 3–6 Mya in southwestern [38] and colonized northwestern Madagascar only during the Late Pleistocene [42].

The present study aims to investigate the impact of the Late Quaternary environmental changes on the demographic dynamics of these two mouse lemur species (*M. murinus* and *M. ravelobensis*) living in partial sympatry in the lowland forests of northwestern Madagascar in spite of their different phylogeographical background. Notwithstanding recent advances in analytical tools to reconstruct populations dynamics of non-model organisms (e.g., [43, 44]), previous studies on lemurs were mostly based on a reduced number of molecular markers (but see [45–47]) or a single demographic method (but see [19, 48]). Also, with the exception of very few studies (e.g., [19, 48, 49]), possible effects of population structure (i.e., non-random mating) on demographic inferences have been largely neglected and results were often interpreted exclusively as size changes of panmictic populations (e.g., [45, 46]). However, it has been shown that population structure can generate spurious signals of population size changes, even when the populations were stationary through time [50–54]. To overcome these limitations, we used two types of genome-wide data (Restriction site Associated DNA sequencing (RADseq) and whole-genome sequences) and three complementary demographic approaches (*Stairway Plot*, *PSMC* and *IICR*-simulations) to model both changes in effective population size (*N*_*e*_) and connectivity over time in *M. murinus* and *M. ravelobensis* in northwestern Madagascar. The *Stairway Plot* [55] and *PSMC* [56] methods have been widely used to detect population size changes. The first typically uses genomic data from independent *loci* obtained from a population sample of several individuals, whereas the second method uses the whole-genome of a single diploid individual. The methods also differ in the fact that the *Stairway Plot* method has been shown to perform best towards the recent past whereas the *PSMC* is more informative about events occurring in a more distant past [55, 57–60]. Results from both methods have been interpreted by assuming that the genomic data used for the analyses stem from a panmictic population. However, theoretical work and simulations have shown that the *PSMC* dynamics observed for many species that is interpreted as reflecting population size changes might also be caused by population structure and changes in connectivity. More specifically, the *PSMC* curves are actually estimates of a complex temporal and sample-based function called the *IICR* (*Inverse Instantaneous Coalescence Rate*). Under panmixia, the *IICR* and the *PSMC* should be interpreted as changes in population size, but the same *PSMC* plot may also have been generated under vastly different modelling conditions, such as a stationary n-island model that undergoes a series of changes in connectivity [53]. For this reason, we also tested the impact of different modeling parameters on the estimated *IICR* in order to evaluate whether the trajectories revealed by coalescent-based methods such as the *PSMC* [56] may also be the result of potential changes in connectivity in a structured population of a constant size [53, 61].

Based on our knowledge about Quaternary climatic oscillations in Africa/Madagascar and in the ecology of our study species, we hypothesize that (I) *M. murinus* should have undergone a founder effect during its relatively recent colonization of northwestern Madagascar and should subsequently have expanded its population size in the region but not during the LGM (hypothesis I); (II) *M. ravelobensis* shows signals of a demographic decline and/or reduced levels of population connectivity during the LGM (hypothesis II); (III) both *M. murinus* and *M. ravelobensis* show signatures of population size increase and/or higher levels of population connectivity during the AHP (hypothesis III); and finally (IV) both mouse lemur species underwent a population decline and/or a decrease in population connectivity after the termination of the AHP (hypothesis IV) (see Table 1). The results of our study will provide a first step towards a better understanding of species responses to past climatic changes in Malagasy lowland forests.

**Table 1:** Summary of the expected demographic dynamics for *M. ravelobensis* and *M. murinus* under population panmixia and population structure

Hypothesis	Climatic event	*M. ravelobensis*		*M. murinus*	
		Panmixia	Structure	Panmixia	Structure
Hypothesis I	Late Pleistocene	_	_	Founder event	_
Hypothesis II	LGM	Bottleneck	↓ MIG	–	–
Hypothesis III	AHP	Expansion	↑ MIG	Expansion	↑ MIG
Hypothesis IV	AHP termination	Bottleneck	↓ MIG	Bottleneck	↓ MIG

The demographic hypotheses are based on available knowledge about past Quaternary climatic dynamics in Madagascar and on the species ecology. LGM = Last Glacial Maximum; AHP = African Humid Period; ↑ MIG = higher migration rate; ↓ MIG = lower migration rate

## Results

### Genomic resources

Both species of mouse lemurs were trapped in two forest sites within the Ankaranfantsika National Park (ANP), Ravelobe and Ankomakoma. Similar to previous studies in the region, the capture data revealed that the two species were not evenly distributed within the two forest sites, even though the trapping effort was the same along all transects [41] (Fig. 1b,c and Additional file 1: Table S1 for details). Two genomic datasets were generated based on the RADseq data for complementary analyses. The Analysis of Next Generation Sequencing Data (ANGSD; dataset 1) [62] pipeline resulted in genotype likelihood information from a total of 324,608 variable sites for *M. murinus* (n = 22) and 601,571 variable sites for *M. ravelobensis* (n = 56). The number of SNPs in the called genotype dataset (dataset 2) yielded 122,053 SNPs for *M. murinus* (n = 22) and 242,121 for *M. ravelobensis* (n = 56). The mean depth per individual ranged between 13.82X and 42.18X for *M. murinus*, and between 13.96X and 47.88X for *M. ravelobensis* (Additional file 1: Tables S2 and S3). Additionally, we generated whole-genome sequences of three female individuals. Whole-genome sequencing and mapping of the single *M. murinus* and the two *M. ravelobensis* individuals resulted in 2,197,400,000 (*M. murinus*), 2,154,480,000 (*M. ravelobensis*, Ravelobe) and 2,159,200,000 (*M. ravelobensis*, Ankomakoma) sites for the *PSMC* analyses. The mean depth of coverage ranged between 16.02X (*M. murinus,* Ankomakoma), 17.04X (*M. ravelobensis*, Ravelobe) and 18.79X (*M. ravelobensis*, Ankomakoma).

### Present day population structure and isolation-by-distance

**Fig. 2:**
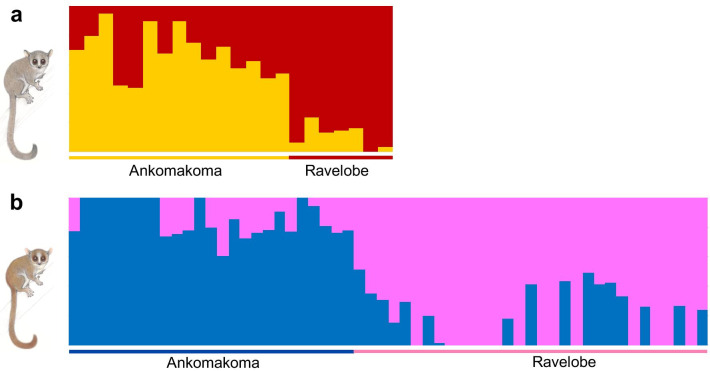
Population genomic structure of the two mouse lemur species. **a** Clustering assignment of 22 M*. murinus* individuals, and **b** Clustering assignment of 56 M*. ravelobensis* individuals to two genetic clusters (K = 2) using dataset 1, respectively. Each single vertical bar represents an individual and each color a distinct genetic cluster. Samples are sorted according to sampling site and respective latitude. Animal illustrations copyright 2013 Stephen D. Nash / IUCN SSC Primate Specialist Group. Used with permission

A weak to moderate genomic signal of population structure was detected in the two mouse lemur species. First, pairwise F_ST_ estimates suggested low levels of genetic differentiation between the two forest sites, although the value was significant for *M. ravelobensis* (*M. murinus*: F_ST_ = 0.011, p = N.S.; *M. ravelobensis*: F_ST_ = 0.015, p < 0.0001). Second, individuals of both species were rather continuously spread along the first axis (> 22% variation explained) of a Principal Component Analyses (PCA), although no overlap existed between both sites (Additional file 1: Fig. S1a, b). Third, a rather fine-scale population-genomic structure was revealed among the two sites for *M. murinus* and *M. ravelobensis* by the NGSadmix analyses, as the admixture plots revealed no complete separation under K = 2 (Fig. 2, but see Additional file 1: Figs. S2 and S3). Instead, substantial levels of admixture were observed in both species and both locations, indicating the occurrence of gene flow between the two close-by sites. Finally, a Mantel test based on all dyadic comparisons revealed a positive and significant correlation between geographical and genetic distance in both mouse lemur species (Mantel statistic r = 0.2344 for *M. murinus* and r = 0.2173 for *M. ravelobensis*, p < 0.001), supporting a pattern of isolation-by-distance.

### Demographic modelling

**Fig. 3:**
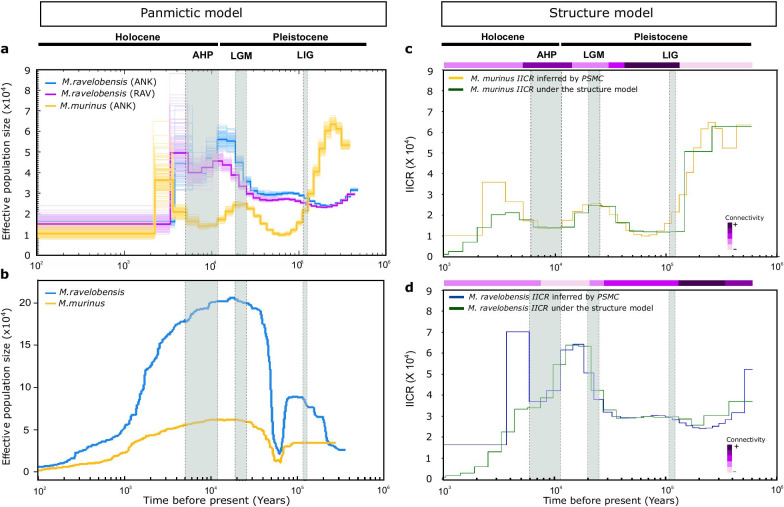
Reconstruction of history of *M. murinus* and *M. ravelobensis* using three complementary methods. The grey vertical bars identify three well-pronounced climatic events in Africa: LIG (Last Interglacial), LGM (Last Glacial Maximum) and AHP (African Humid Period). All analyses were performed considering 2.5 years as generation time and 1.2 × 10^–8^ as mutation rate. **a** Demographic history inferred by the *PSMC* method using “4 + 25*2 + 4 + 6” free atomic time intervals. The thick lines represent the inferred mean trajectories for three populations, and each light line represents 100 subsampled bootstrap replicates for each individual. The humps observed in *PSMC* plots of the two mouse lemur species during the last 2–5 kyr seem to be a common artefact (e.g., see [6, 56]) and do not correspond to a real demographic event. Note that those humps are no longer present when we consider a different free atomic time interval (see Additional file 1: Fig. S6). **b** Reconstruction of the demographic history of *M. murinus* (N = 22) and *M. ravelobensis* (N = 55) using the *Stairway Plot* method, considering the two forest sites together. **c** and** d** *PSMC* plots estimated from the genomic data and *IICR* obtained from simulated data for both species assuming (i) a n-island model of migration; (ii) a constant population size over the time, (iii) the occurrence of five changes in population connectivity. The color code for the top horizontal bars summarizes the inferred changes in connectivity across time for each species. According to the simulation results, population connectivity changed in *M. murinus* at ~ 129.1 kyr (LIG), 42.7 kyr, 30.7 kyr, 13.7 kyr (onset of AHP) and 5.1 kyr (termination of AHP, Fig. 3c). Accordingly, population connectivity changed in *M. ravelobensis* at ~ 338.9 kyr, 135.6 kyr (LIG), 27.1 kyr, 20.1 kyr (LGM) and 7.9 kyr (Fig. 3d). RAV = Ravelobe; ANK = Ankomakoma

The demographic history of *M. murinus* and *M. ravelobensis* was inferred using three complementary approaches (*Stairway Plot, PSMC, IICR*-simulations). The *Stairway Plot* [55] and the *PSMC* (Pairwise Sequentially Markovian Coalescent, [56]) were used to infer population size changes from the RADseq data and whole-genome sequences, respectively. The *Stairway Plot* analyses with the entire dataset for each forest site (Additional file 1: Fig. S4) and for the two sites together (Fig. 3b) suggested the same overall demographic trend for *M. murinus* and *M. ravelobensis*. Both species exhibited an increase of population size < 100 kyr and unexpectedly reached their largest size between the LGM and the African Humid Period (N_e_ ~ 210,000 for *M. ravelobensis* and ~ 60,000 for *M. murinus*). These maxima were followed by a subsequent continuous decline in population size that started at the onset of the AHP and lasted until the present. *Stairway Plot* results consistently suggested a higher *N*_*e*_ for *M. ravelobensis* than for *M. murinus*, even after standardizing the number of individuals in the analyses (n = 7 per forest site and species, Additional file 1: Fig. S5). Despite the sex-biased dispersal patterns suggested for our study species [63–65], the *Stairway Plot* analyses performed with the *M. ravelobensis* dataset (n = 22 males and 22 females) confirmed an identical demographic history when considering males and females separately (Additional file 1: Fig. S6).

The *PSMC* suggested a similar demographic trend for *M. ravelobensis* as the previous method, but different dynamics for *M. murinus* (Fig. 3a). Specifically, it inferred a rather stable population size of *M. ravelobensis* between the LIG period (~ 130 kyr) and about 30 kyr which was followed by a population increase that reached its maximum around the onset of the AHP in Madagascar (~ 15 kyr; [18]; *N*_*e*_ ~ 45,000 for Ravelobe and ~ 55,000 for Ankomakoma). Population sizes then decreased towards the present. The recent peak observed in both curves could be interpreted as an increase of population size. However, this peak was no longer present in the *PSMC* when using a different free atomic time interval (Additional file 1: Fig. S7). Therefore, the recent peaks most likely represent a common artefact (also present in other studies; e.g., see [6, 56]) rather than a real demographic event.

The *PSMC* for *M. murinus* suggested an ancient massive population decline which started before the LIG period and reached a minimum at ~ 70 kyr (*N*_e_ ~ 10,000). This bottleneck was followed by a population recovery that reached an unexpected moderate maximum during the LGM (19–26.5 kyr, *N*_e_ ~ 25,000). Afterwards, *M. murinus* experienced a second population decline that lasted until the AHP. This event was followed by a population size increase until ~ 3 kyr that may be partly, but not entirely, due to the above mentioned artefact, and a subsequent population decline towards the present (see Additional file 1: Fig. S7).

The previous interpretations of the *PSMC* rely on the assumption that population structure can be neglected and that individuals were part of panmictic populations. Alternatively, it is possible that mouse lemur populations are structured into sub-populations (= islands) and that changes in the *PSMC* are caused by changes in migration rate between them over time (= changes in connectivity). As a first approximation we estimated the effects of connectivity changes in a n-island model under constant population size using simulations of the *IICR* (Inverse Instantaneous Coalescence Rate) as in [61]. These simulations suggested that models with a large number of islands (n = 61 for *M. ravelobensis* and n = 84 for *M. murinus*) and five major historical changes in population connectivity per species may best explain the dynamics inferred for the *IICR* (Fig. 3c, d). The congruence between the two *IICR* curves inferred by the *PSMC* and by simulations is relatively high for the dynamics older than 7 kyr in both species. The timing of the changes in population connectivity overlapped only partly between the two mouse lemur species (horizontal bars above curves in Fig. 3c, d). In both species, population connectivity appeared to have been higher during and after the LIG than during the LGM. However, whereas population connectivity stayed relatively low for *M. ravelobensis* across the last 30 kyr, population connectivity was again higher during the AHP for *M. murinus*, the species thought to have undergone a recent expansion into northwestern Madagascar [42].

## Discussion

### The baseline: present-day population structure and connectivity among forest sites

The relatively low levels of genetic differentiation and the relatively high levels of genomic admixture between sampling sites suggest the existence of gene flow and therefore genetic connectivity among the two sampling sites in both mouse lemur species at present times. Ravelobe and Ankomakoma are separated only by about 10 km straight-line distance, but some larger patches of savannah would preclude straight-line dispersal between them (e.g., [42, 66]). Although the two sub-populations showed an isolation-by-distance effect, our results suggest that the two forest sites were still connected via some forest corridors that surrounded the savannah (see Additional file 1: Fig. S8) and may facilitate the dispersal of small organisms such as mouse lemurs [67]. Nevertheless, dispersal events are likely limited to small geographical distances, and should by all means be smaller than the geographic distance between both sites, since dispersal distances of mouse lemurs were previously shown to reach a maximum of up to 1 km (*M. murinus* in western Madagascar; [68] but see also [66, 69, 70]). Furthermore, multiple genetic studies showed that most dispersal events in mouse lemurs (e.g., *M. murinus; M. ravelobensis*; *M. tavaratra*) [64, 66, 68–71], but also in larger body sized lemur species with longer dispersal distances (e.g., *Propithecus tattersalli* and *P. perrieri*) [72, 73] occur among neighboring social groups. Altogether, our results suggest that mouse lemurs from both forests are part of a larger set of sub-populations that were connected, and maybe still are.

### Taking stock: historical ecological changes inferred for northwestern Madagascar

The longest paleoenvironmental record available for Madagascar stems from a sediment core from Lake Tritrivakely in the central highlands (154 kyr) [32]. This record is the only one from the island reaching back to the Last Interglacial, and suggested that the vegetation of the highlands during this period was dominated by a grassland/woodland mosaic. This vegetation was replaced by Ericaceae (adapted to survive strong seasonal droughts) [31, 32] during the LGM as a result of the abrupt decrease in temperature (~ 4 ℃ cooler than today) [31, 74]. Multiple paleoenvironmental records from other parts of the island and corresponding to more recent periods confirmed substantially drier and cooler conditions during the LGM (e.g., [18, 30, 75, 76] but see [77]), suggesting that a general contraction of forest habitats across Madagascar at that time period is likely [30]. Such conditions during the LGM probably induced lowland forests to contract, possibly to riverine refugia, as those areas would have secured access to water and generated rather mesic local conditions [33]. As a consequence, many forests would have become partly isolated or only connected via mosaic forest corridors. This period was followed by an abrupt warming and increasing levels of moisture across the island, largely in congruence with the African Humid Period in continental Africa [17, 18, 31, 32]. A paleoenvironmental record from the Anjohibe Cave in northwestern Madagascar confirmed that the climatic conditions were wetter in the early-Holocene (9.1–4.9 kyr; AHP). It can therefore be expected that the wetter conditions favored the expansion of the woodland/grassland vegetation in the Malagasy lowland forests [25]. Finally, the period that followed the AHP was marked by a climatic warming and aridification in northwestern Madagascar, resulting in a vegetation shift towards a grassland-dominated landscape at ~ 4.8 kyr [17]. This ecosystem shift was likely intensified by the introduction of swidden agriculture and spread of pastoralism in the region during the last two millennia [17, 75, 78].

### Demographic history of *M. ravelobensis*

Assuming that population structure is negligible, the *PSMC* and *Stairway Plot* inferred a population expansion of *M. ravelobensis* starting before the LGM, whereas the *IICR-*simulations under population structure suggested that the same *PSMC* dynamics could be the result of a decreased population connectivity under constant population size. Considering the presumably rather cold and arid environmental conditions in northwestern Madagascar across the LGM [30, 33], a population expansion was rather unlikely. However, a decrease in connectivity between sub-populations of *M. ravelobensis* would be concordant with the hypothesized contraction of forests during this period, since animal populations in the resulting mosaic landscapes would be less connected than before (hypothesis II; see introduction).

The *PSMC* and *Stairway Plot* suggested that *M. ravelobensis* subsequently underwent a demographic decline that started before the onset of the AHP. Such a scenario is also rather unlikely, given that lowland forests were presumably at their maximum extension at these times due to warmer temperatures and sufficient water supply [14, 15]. Our *IICR*-simulations suggested that the same *PSMC* curve could be the result of low levels of population connectivity for *M. ravelobensis* during the AHP. This scenario also contradicts our predictions for mouse lemur populations during the AHP (hypothesis III). Moreover, they are not in congruence with the ecological preference of *M. ravelobensis* for mesic microhabitats that should have been widespread during this time (e.g., [41, 79]). One possible explanation for these conflicting results could be the potential interspecific competition with *M. murinus* after its expansion into an inter-river system that was previously only inhabited by one mouse lemur species, *M. ravelobensis* [40]. Previous field studies confirmed that *M. murinus* has a higher competitive potential than *M. ravelobensis* in an experimental setting [80]. The spatial expansion of *M. murinus* may have required new patterns of habitat partitioning and resulted in new direct or indirect interspecific competition (e.g., for food resources, sleeping sites). These species interactions may have precluded establishing large population sizes and/or higher population connectivity for *M. ravelobensis* during the AHP.

The *PSMC* and *Stairway Plot* inferred a continuing population decline for *M. ravelobensis* after the termination of the AHP, which would be in concordance with hypothesis IV. The increasing aridification during the mid-Holocene and the ecosystem shift towards a grassland-dominated landscape [17, 75, 78] might have contributed to this development. Such a decline was already inferred in previous studies on this and other lemur species inhabiting the northwestern region (e.g., *Microcebus bongolavensis, M. danfossi*, and *Lepilemur edwardsi*) [81, 82] and in multiple Malagasy species distributed across the island (e.g., frogs, birds of prey and rodents) [83–85] and was mostly attributed to intensified human pressures on the island during the last thousand years. It should be noted that the *IICR*-simulations did generate the same dynamics under a model of population structure and constant population size. This approach required similar population connectivity levels during recent times than in the LGM. Such reduced levels of population connectivity would be congruent with the loss of suitable mouse lemur habitats across the late-Holocene [17, 75, 78]. In conclusion, it appears that both changes in population connectivity (LGM) and size (late-Holocene) may have happened and shaped the demographic dynamics of *M. ravelobensis* populations over time.

### Demographic history of *M. murinus*

The late Pleistocene demographic dynamics of *M. murinus* were expected to be shaped by its rather late colonization of northwestern Madagascar [42]. Such a colonization would be a classical example of a founder effect with a relatively small number of individuals arriving in this region via a highland corridor [86, 87]. Such an event would very likely mirror a population bottleneck and would have been followed by a rapid spatial and demographic expansion into the lowland forests between the Betsiboka and Mahajamba rivers (hypothesis I). Our demographic analyses, if interpreted in terms of population panmixia, indeed suggested that *M. murinus* underwent a strong population bottleneck at around 70 kyr, and that the effective population size (*N*_*e*_) subsequently increased and reached a maximum during the LGM. In fact, our time estimate for the founder effect (~ 70 kyr) does agree very well with that of the previous study (26.5–33.5 kyr) [42] if taking into account that the generation time used in both studies differed by the factor 2.5 (2.5 years [this study, 18, 36] versus 1 year [42]; see methods for details on generation time). However, the *IICR*-simulations revealed that the *IICR* dynamics may also have been the result of changes in population connectivity under stable population size. Indeed, the *IICR*-simulations suggested higher levels of population connectivity during and after the LIG than during the LGM, which would be in concordance with the predicted forest contractions during the LGM [30, 33]. These two competing interpretations cannot be easily reconciled, since both scenarios do partially fit hypothesis I for this species. Information about the definite time point of colonization by *M. murinus* or details of the paleoenvironmental dynamics in this inter-river-system back to the LIG are needed to evaluate the two alternative scenarios.

Towards more recent times and in contrast to our expectations, the *PSMC* and *Stairway Plot* suggested that *M. murinus* underwent a temporary demographic decline that started before the onset of the AHP. Conversely, the *IICR-*simulations point towards higher connectivity levels during the AHP under constant population size, which would be in line with hypothesis III. Assuming that increasing humidity after the LGM likely resulted in an expansion of dry deciduous forests [17], higher levels of gene flow among sub-populations would be rather expected, and were previously documented for *M. arnholdi* in northern Madagascar [18].

Finally, both *PSMC* and the *Stairway Plot* revealed a population decline for *M. murinus* during the late-Holocene (hypothesis IV), which is congruent with the climatic warming and aridification that followed after the AHP [17] and the presumably increasing degree of habitat fragmentation in the region [17, 75, 78]. The timing of the *M. murinus* population decline coincided well with previously documented bottlenecks for other Malagasy species and with the collapse of the Malagasy megafauna (< 3 kyr; e.g., [25, 30, 88]).The lower levels of population connectivity during recent times suggested by the *IICR*-simulations would also be in concordance with the hypothesis IV.

## Conclusions

The present study suggests that climatic fluctuations have been important drivers of evolutionary trajectories for mouse lemurs in northwestern Madagascar. Interestingly, our demographic reconstructions also revealed distinct dynamics for *M. murinus* and *M. ravelobensis*, suggesting that even closely related species may differ in their responses to the same climatic events. Different demographic scenarios emerged for both mouse lemur and the decision for one of possible alternative explanations was not always straight forward. Population structure and changes in connectivity very likely impacted the demographic dynamics of mouse lemurs in this region of Madagascar (see Additional file 1: Table S4), while forest contractions and expansions may have extensively shaped the history of lowland forests during the Late Pleistocene (e.g., [33]) like in other regions of the world (e.g., [89–93]). For example, a decrease in population connectivity may explain the *IICR* dynamics during the LGM better than population size changes in both species. However, it also became clear that changes in connectivity alone may not explain well all findings. For instance, if the *PSMC* dynamics before the LGM would be the result of changes in connectivity alone, this would imply that *M. murinus* must have colonized northwestern Madagascar already earlier than the LIG, because a founder event and subsequent colonization of this IRS would necessarily include population size changes. Systematic simulations are needed to clarify the consequences of a founder event and a subsequent spatial expansion on the *IICR* dynamics when considering a n-island model of migration.

The two alternative models studied here represent endpoints on a scale of possible events, and mixed scenarios including both population size and connectivity changes may ultimately explain best the historic population dynamics of mouse lemurs in northwestern Madagascar (e.g., see [18, 19]). Therefore, the implementation of model-based approaches such as the Approximate Bayesian Computation [94] or composite-likelihood methods [95] is crucial to test and compare alternative demographic hypotheses including both population size and connectivity changes (e.g., [19, 96, 97]). In addition, further high-resolution paleoenvironmental reconstructions for northwestern Madagascar are urgently needed to better understand the impact of the last Interglacial–Glacial cycle on the lowland dry forest dynamics. Altogether, our study shows that it is essential to consider the impact of different model assumptions (e.g., panmixia and population structure) when exploring, weighting and inferring alternative species demographic scenarios. In particular, the application of the *PSMC* should in the future be always complemented with a simulation approach in order to avoid oversimplification.

## Methods

### Study species

The sympatric *M. murinus* and *M. ravelobensis* display marked differences in their distribution, evolutionary history and ecology. It was previously estimated that *M. murinus* and *M. ravelobensis* diverged by about 9.60 Ma [98], although diversification dates for mouse lemurs were recently challenged [47]. While *M. murinus* is regarded as a habitat generalist due to its wide distribution across various forest types in western and northwestern Madagascar (reviewed in [39]), *M. ravelobensis* is assumed to be more specialized, although it was shown to reach higher local abundancies and a broader distribution within the Ankarafantsika National Park than its congener [41]. Both species occur in partial sympathy in northwestern Madagascar but were shown to respond differently to habitat fragmentation [79]. While *M. murinus* can be often found in even small forest fragments and seems to have a higher overall vagility across mixed and partially open landscapes, *M. ravelobensis* is typically found in larger forests and may be less able to connect across open landscapes [79]. Both species were shown to have different microhabitat preferences [41, 99], and direct competition between the two species is therefore not regarded as main mechanism of abundance regulation [41, 79]. Both species forage solitarily during the night, but form sleeping groups during the day that vary in composition between the species. *M. murinus* males sleep alone and females form groups of related individuals in wooden tree holes, while *M. ravelobensis* forms mixed‐sex sleeping groups consisting of matrilinear relatives of varying degree of relatedness who use diverse substrates as sleeping site (e.g., lianas and leaves) [65, 100, 101]. Natal dispersal in *M. murinus* is male-biased [63, 64], whereas *M. ravelobensis* displays only moderate and delayed male-biased dispersal which was suggested to lead to a higher risk of inbreeding [65].

### Study area and sample collection

The Ankarafantsika National Park (ANP) is located in northwestern Madagascar, in an area of about 135,000 ha of dry deciduous forest that is delimited by the Betsiboka (western limit) and the Mahajamba rivers (eastern limit; IRS Ia; Fig. 1a) [40, 41]. The ANP is one of the largest remaining forest blocks in western Madagascar [42], although it shows some degree of forest fragmentation towards the edges [41, 79]. The locally sympatric *M. murinus* and *M. ravelobensis* were sampled around two natural lakes on the western part of the ANP, Ravelobe (Rav, –16.302413°N, 46.821346°E) and Ankomakoma (Ank, -16.342752°N, 46.740293°E, Fig. 1b, c). The sites are approximately 10 km apart and characterized by a mosaic of savannah and forest corridors. Ravelobe (85–176 m above sea level, a.s.l.) is situated near Ampijoroa next to the National Road RN4 that connects Antananarivo to Mahajanga, while Ankomakoma (105–185 m a.s.l.) is part of a mosaic landscape consisting of dry deciduous forest with interspersed savannah patches (Additional file 1: Fig. S8).

Fieldwork took place during the dry season of 2017 (April–July). Four 1 km transects were installed for field work in each site in order to cover various forest parts around the lakes. All transects followed pre-existing dirt roads or foot paths. A total of 1200 Sherman Traps (Sherman Traps Inc, Tallahassee, FL, USA), baited with banana, were installed overnight in Ravelobe across 12 nights, and 1000 traps were installed in Ankomakoma across 10 nights (see [102] for details). *M. murinus* and *M. ravelobensis* were distinguished in the field based on their head coloration (greyish in *M. murinus* vs. brownish in *M. ravelobensis*) [103] and their distinctive tail length (130.81 ± 6.15 mm in *M. murinus* vs. 155.48 ± 7.57 mm in *M. ravelobensis*) [40]. All animals were released at dusk of the same day at their individual capture position. Small ear biopsies (approx. 2–3 mm^2^) were taken from all captured animals for genomic analyses. Tissue samples were stored in Queen’s lysis buffer [104] during the field season and subsequently at − 20 °C in the laboratory.

### RADseq library & whole-genome sequencing

A total of 24 M*. murinus* (7 from Ravelobe and 17 from Ankomakoma) and 60 M*. ravelobensis* (33 from Ravelobe and 27 from Ankomakoma) individuals were available for RADseq, based on the spatial distribution and abundance of each mouse lemur species per transect and sampling site. Total genomic DNA was extracted from the ear biopsies using the DNeasy Blood & Tissue Kit (Qiagen) following the manufacturer’s protocol with few modifications (see [46] for details), and the DNA concentration was estimated with the Qubit® Fluorometer (Life Technologies). DNA samples (~ 200 ng of DNA) were then digested with the restriction enzyme SbfI at the GenoToul-GeT-PlaGE Core Facility (Toulouse, France). RAD Libraries were prepared in sets of 24 samples sorted by original DNA concentration, where each sample was assigned to one of 48 unique barcode sequences during adapter ligation. Sub-libraries were randomly sheared [105, 106] using a Covaris M220 ultrasonicator, resulting in fragments with an average size of 550 bp. Sheared DNA fragments were ligated to the second adapter and all fragments with both adapters were amplified in 10 Polymerase Chain Reaction (PCR) cycles. DNA concentration and fragment sizes of the amplified libraries were verified using qPCR and Fragment Analyzer. The sub-libraries were sequenced using 150 bp paired-end reads on an Illumina HiSeq3000 platform at the GenoToul-GeT-PlaGE Core Facility (Toulouse, France). Raw data was initially demultiplexed using the tool *splitbc* implemented on the FASTX-toolkit (http://hannonlab.cshl.edu/fastx_toolkit/), and the quality of the raw data was verified with FastQC v0.11.7 (http://www.bioinformatics.bbsrc.ac.uk/projects/fastqc). Trimmomatic v0.36 [107] was used to remove Illumina adapters from the reads, remove reads with low quality bases, and to trim the reads to a minimum 4-base sliding window with quality score below 15. Additionally, reads with less than 60 bp length after the filtering steps were removed from the analyses. After quality filtering, BWA-MEM (http://bio-bwa.sourceforge.net/) was used to align the paired-end reads to a high quality genome assembly of *Microcebus murinus* (GCA_000165445.3) [108]. Finally, the software SAMtools v1.8 [109] was used to remove PCR duplicates (i.e., sequence reads that result from sequencing two or more copies of the exact same DNA fragment; [110]) and to convert the Sequence Alignment Map (SAM) format to the corresponding binary version (BAM). To ensure that only autosomal data was used for the analyses, the *M. murinus* scaffold NC_033692.1 (designated as the X-chromosome) as well as the *M. murinus* mitochondrial genome (NCBI Accession Number: KR911908.1) were excluded from the aligned BAM files. The autosomal BAM files were then used as input files for all downstream genomic analyses.

In addition to the RADseq dataset, one *M. murinus* (Ankomakoma) and two *M. ravelobensis* samples (Ravelobe and Ankomakoma) were selected for whole-genome sequencing. All samples were females which are the philopatric sex in both species [65, 69]. Given that no female *M. murinus* was caught at Ravelobe, no whole-genome sequence was generated at this site for that species. Libraries were prepared at the Institute for Animal Breeding and Genetics of the University of Veterinary Medicine Hannover using the NEBNext Ultra DNA Library Prep Kit from Illumina (New England BioLabs, Ipswich, MA, USA). DNA samples (~ 200 ng of DNA) were first sheared with an ultrasonicator (Covaris M220, Woburn, Massachusetts, USA) and the respective sizes were selected according to the manufacturer’s recommendations. Whole-genome sequencing was performed on an Illumina NextSeq 500 (Illumina, San Diego, CA, USA) for 300 cycles in paired‐end mode. Visual quality control of whole-genome sequencing data was performed using FastQC version 0.11.7. Reads were trimmed using PRINSEQ version 0.20.4 [111] and mapped to the *M. murinus* reference genome (“Microcebus_murinus.Mmur_3.0.dna.toplevel.fa.gz”) using the BWA-MEM algorithm implemented in the BWA version 0.7.17 [112]. Similarly to the RADseq dataset, the reads that mapped against the *M. murinus* X-chromosome and mitochondrial genome were discarded from our analyses (see details above). For all analyses with the whole-genome sequences, the read depth and quality of the variant sites were controlled by applying the following quality filters: base quality above 20, mapping quality above 30, minimum read depth of 3 and a maximum read depth of 100. See Additional file 1: Text S1 and S2 for details about the sequencing libraries.

### RADseq datasets: genotype likelihoods & SNP calling

Next-Generation Sequencing platforms can generate large amounts of sequencing data but are prone to sequencing errors [113–115]. It is therefore advisable to keep the data in form of genotype likelihoods during downstream analyses, because genotype likelihoods retain information about uncertainty in base calling, which enable to control for some problems commonly associated with RADseq datasets (e.g., unevenness in sequencing depth and allelic dropout) [114, 116, 117]. Consequently, most of our analyses were carried out on genotype likelihoods estimated with ANGSD [115], considering the following filters: a minimum base quality of 20, a minimum mapping quality of 30, minimum Minor Allele Frequency below 0.5, a minimum mean depth of coverage of 4X [115, 117, 118], and sites present in at least 75% of the individuals (dataset 1: genotype likelihoods). In addition to the genotype likelihoods, genotypes were called for each mouse lemur species for complementary analyses. Genotypes were called with SAMtools v1.8 [109] using the same quality filters previously used in ANGSD (see above), but considering a minimum depth of coverage of 10× to ensure high-confidence genotype calls (dataset 2: genotype calls) [119].

Knowing that population genetics analyses can be biased by social structure and relatedness between individuals (e.g., [120, 121]), a relatedness analyses was performed next. Relatedness between two individuals is usually described by the concept of identity-by-descent (IBD), where two alleles are considered identical by descent if they recently descended from a common ancestral allele [117, 122]. NGSrelate [117] was used to calculate the IBD coefficients (k_0_, k_1_ and k_2_; probability of two individuals sharing 0, 1 or 2 alleles from a single ancestor at any locus, respectively) between pairs of individuals using dataset 1 [122]. First-degree relatives were then inferred based on the comparison of the obtained IBD coefficients with the expected IBD probabilities (i.e., parent-offspring: k_0_ = 0, k_1_ = 1 and k_2_ = 0; full sibs: k_0_ = 0.25, k_1_ = 0.50 and k_2_ = 0.25) [122]. Only one individual of each dyad of closely related individuals was retained in our dataset for downstream analyses (see Additional file 1: Text S3).

### Population-genomic structure & isolation-by-distance

Population-genomic structure patterns were investigated in *M. murinus* (n = 22) and *M. ravelobensis* (n = 56) using three distinct approaches. First, genetic differentiation between both sites was estimated per species using Wright’s F-statistics F_ST_ [123]. The analyses were performed with ARLEQUIN [124] and significance was tested using 10,000 permutations (dataset 2). Second, signals of population genetic structure were evaluated by Principal Component Analysis (PCA) computed using PCAngsd (dataset 1) [125]. The PCA Eigenvalues that explained most of the genetic variation between individuals (PC1 and PC2) were extracted and individuals were plotted using R (R CoreTeam 2014). Third, NGSadmix [114], a maximum-likelihood clustering method, was used to assign individuals to a specific number of clusters (K) using dataset 1. For both species, the number of explored clusters ranged between 1 and 3, and a total of 10 independent runs were performed for each value of K. The most suitable value of K was determined with Clumpak [126], following the Evanno method [127].

Geographically restricted gene flow results in a significant correlation between genetic and geographic distance, known as isolation-by-distance [128], where the genetic dissimilarity increases with the increase of geographical distance [67]. The effect of geographic distance on genomic differentiation over our small geographic scale was investigated using the individual as the unit of the analyses [71]. Genetic dissimilarity between any two individuals was measured by the Rousset’s genetic distance (â) [129], an estimator analogous to the F_ST_/(1-F_ST_) ratio using pairs of individuals instead of populations. Geographic distance was measured as the linear geographical distance in km separating each pair of individuals. Both Rousset’s genetic distance and geographic distance were computed with SPAGeDi [130] based on dataset 2. The occurrence of isolation-by-distance was finally investigated with a Mantel test [131] using the VEGAN package [132] available in R (R Development Core Team 2005). Significance was determined via 10,000 permutations.

In addition, genetic summary statistics were calculated for each species and forest site (see Additional file 1: Text S4 and Table S5 for details). Inbreeding coefficients per individual (*F*) were also estimated using dataset 2 to detect deviations from Hardy–Weinberg equilibrium (Additional file 1: Tables S6 and S7) [133]. One *M. ravelobensis* individual captured at Ravelobe showed a highly negative *F* value and was therefore excluded from the demographic analyses (Additional file 1: Table S6) [133]. For details about the set of individuals used in each step of this study see Additional file 1: Table S8.

### Mutation rate and generation time

The demographic history of both species was investigated using three complementary modeling approaches: *Stairway Plot* [55], *PSMC* [56] and *IICR*-simulations [53]. All analyses were performed assuming a mutation rate value of 1.2 × 10^–8^ [47, 134]. This mutation rate is the most accurate estimate available for mouse lemurs and was calculated from average pedigree-based estimates of seven primates species [47, 135]. During the last decade, generation time (GT) values between 1 and 4.5 years have been used for genetic studies on mouse lemurs [36, 40, 134]. Since a recent study on free-living *M. murinus* from the ANP estimated a 2.5 year generation time as the average age of parents [36], we recently compared the performance of three GT values (1, 2.5 and 4.5 years) for another mouse lemur species (*M. arnholdi*) using alternative demographic methods. As the results under 2.5 years fitted best to on-site high-resolution paleoenvironmental reconstructions [18], we decided to also use this estimate for *M. murinus* and *M. ravelobensis* in this study.

### Stairway plot

The *Stairway Plot* [55] method uses the Site Frequency Spectrum (SFS; i.e., the distribution of the allele frequencies of a given set of SNPs in a population) [136] from population genomic sequence data to estimate a series of population mutation rates (θ = 4N_e_µ) following a multi-epoch demographic model, where epochs coincide with coalescent events [55]. The realSFS tool implemented in ANGSD [115] was used to estimate a folded one-dimensional SFS for each mouse lemur species (i.e., considering the two forest sites together) based on dataset 1, because we lack a suitable outgroup to determine the ancestral state of each allele [136]. The folded 1d-SFS of each species was used as input data to generate 199 additional SFS by bootstrap, following the software guidelines [55]. Inferences were then made based on the 200 1d-SFS for each species with *Stairway Plot* v2.0 [55]. Given the observed isolation-by-distance pattern in both *M. murinus* and *M. ravelobensis*, the *Stairway Plots* were also repeated considering the two forest sites separately. Knowing that changes in *N*_*e*_ through time are dependent on the number of possible coalescent events, this method is sensitive to the number of individuals and SNPs in the dataset [59, 60, 137]. Therefore, analyses were first performed considering the entire dataset of each forest site and species (n ranging between 7 and 30), and also repeated with an equal sample size (n = 7) per site and species. For this analysis individuals of each site were randomly selected using R (R Development Core Team 2005). Finally, to evaluate the effect of sex-biased dispersal in the demographic inferences of mouse lemurs, we reran the *Stairway Plot* for the *M. ravelobensis* dataset considering males and females separately. The analyses were performed considering all *M. ravelobensis* females available in our dataset (n = 22) and an equal number of males to avoid differences in *N*_*e*_ related to sample size. The males were randomly selected using R (R Development Core Team 2005).

### *PSMC* & *IICR*-simulations

The *PSMC* [56] method makes use of the distribution of heterozygous sites along the entire genome of a single diploid individual to estimate the population size change history that best explains the corresponding coalescence times. As shown by [53] the *PSMC* method is actually inferring the *IICR* of the sampled individual. This *IICR* can be interpreted as a history of population size change under panmixia, but under structured models it is more difficult to interpret. Here we focused on the interpretation of *PSMC* plots under either panmixia or piecewise-stationary n-island models. In the latter, we allowed gene flow to be constant for certain periods of time and to change between periods of time that we call components, always assuming that the population structure can be approximated by an n-island model. For instance, if we assumed that there were three components this means that there were three period of time with respective migration rates M_1_, M_2_ and M_3_, and two times at which the migration rates changes, namely t_1_ and t_2_ (t_0_ is assumed to be the most recent time of sampling, i.e., t_0_ = 0). The three whole-genome autosomal sequences (one *M. murinus* and two *M. ravelobensis*) generated as part of this study were submitted to *PSMC* analyses, considering the following parameters: minimum read depth per site of 3 (-d3), maximum read depth per site of 100 (-D100), upper limit of the TMRCA and initial θ/ρ value of 5 (-t5 and -r5). The *N*_*e*_ was inferred using 4 + 25*2 + 4 + 6 free atomic time intervals, with a total of 100 bootstrap replicates (command line: ‐N30 –t5 –r5 –p “4 + 25*2 + 4 + 6” ‐D100 –d3 –q30). The PSMC analyses were repeated considering the same parameters but using -p “64*1” free atomic time intervals. Since the mean genome coverage of two of our sequences was close to but below the recommended 18X (see [6]), simulations for variant coverage divergence were performed to evaluate the impact of lower genome-wide coverages in the *PSMC* inferences for mouse lemurs. The analyses were performed for the *M. ravelobensis* individual sampled in Ankomakoma by varying the minimum read depth option per site (-d) between one and twelve. Results are discussed in the Additional file 1: Text S5 and Fig. S9.

In order to find a demographic scenario that could explain the observed *PSMC* curves under a piecewise stationary n-island model as mentioned above, we used the *SNIF* (Structured Non-stationary Inference Framework) inferential method of [138]. This method uses the *PSMC* plot as a summary statistic of genomic information and as a target for an optimization algorithm under a piecewise stationary n-island models with unknown parameter values (N, n, t_i_, M_i_), where N is the deme size, n is the number of demes, and the t_i_ values correspond to the times at which the migration rates M_i_ change. *SNIF* uses a search algorithm that explores the parameter space and uses an optimality criterion to select the structured scenario that best explains a given target *IICR* (simulated) or *PSMC* (observed or simulated) curve. The method has been validated using target *IICR*s generated under piecewise stationary n-island models of increasing complexity (i.e., number of components or periods during which the M_i_ can change) by comparing inferred and simulated parameter values. Technical details of the search algorithm and distance computation can be found in [138]. Here we simply note that the method finds the parameter values that minimize a distance between the *IICR* curve generated under a very large number of piecewise stationary n-island models, and the observed *PSMC*. The algorithm stops its search when it reaches a minimum distance set by the user or a pre-set number of optimization steps. The final scenario is then validated by generating an *IICR* curve under these "best parameter values" and by running *SNIF* on this *IICR* to test whether the method would indeed re-infer the same values. If *SNIF* does not manage to infer the right scenario, this suggests that the scenario is not to be trusted as even if it were correct the method would not infer it properly. If we infer the right parameters, this suggests that the scenario is inferable by our method, but it cannot be seen as a proof that it is correct. This is the best scenario under the piecewise stationary n-island model that (i) explains the data and (ii) is of reasonable complexity and (iii) can be inferred and thus trusted to some extent. In order to produce a well estimated *IICR* curve we simulated 100,000 T2 values for the *M. murinus* and the *M. ravelobensis* individuals sampled in Ankomakoma using the ms software [139]. The *IICR* was plotted with the observed *PSMC* using a python script available at: https://github.com/willyrv/IICREstimator [61]. The SNIF program and its documentation can be found in github.com/arredondos/snif. The ms commands used to generate the best-fitting *IICR* plots can be found in the Additional file 1: Text S6.

### Impact of repeat regions in the demographic modelling

It has been recently shown that genomic repeat regions may affect demographic inferences using diverse methods, including the *Stairway Plot* and *PSMC* [140]. To evaluate the impact of repeat regions in our demographic inferences, we reran the *Stairway Plot* for the entire *M. ravelobensis* dataset (n = 55) and the *PSMC* analyses for the three whole-genome sequences without the repeat regions. We firstly removed all repeat regions from our BAM files following the RepeatMasker output file kindly provided by J. Rogers for the *Microcebus murinus* reference genome (GenBank Assembly Accession Number: GCA_000165445.3 [108]). We then reran the *Stairway plot* and *PSMC* using the same command options aforementioned. Results are presented in the Additional file 1: Text S7 and Fig. S10.

## Supplementary Information

**Additional file 1.** Supplementary text (Text S1–Text S7), figures (Figure S1–Figure S10) and tables (Table S1–Table S10).

## Data Availability

The RADseq sequences generated under this study are publicly available at Sequence Read Archive (NCBI) in the BioProject PRJNA560399 (Number Accession: SAMN16955525–SAMN16955602). Whole-genome sequences are available in the BioProject PRJNA632451. Accession Numbers: SAMN15237929 (*M. ravelobensis*, Ravelobe), SAMN15246264 (*M. ravelobensis*, Ankomakoma), SAMN15237931 (*M. murinus*, Ankomakoma). Scripts used for all analyses are available upon reasonable request.

## Abbreviations

a.s.l.: Above sea level
AHP: African Humid Period
ANK: Ankomakoma
ANP: Ankarafantsika National Park
IBD: Identity-by-descent
IICR: Inverse Instantaneous Coalescence Rate
IRS: Inter-River-System
kyr: Thousand years
LGM: Last Glacial Maximum
LIG: Last Interglacial
MIG: Migration rate
M.mur: *M. murinus*
M.rav: *M. ravelobensis*
Mya: Million years ago
N_e_: Effective population size
PCA: Principal component analyses
PSMC: Pairwise Sequentially Markovian Coalescent
RADseq: Restriction site Associated DNA Sequencing
RAV: Ravelobe
SFS: Site frequency spectrum
