# Supplementary material for: Impact of model assumptions on demographic inferences: the case study of two sympatric mouse lemurs in northwestern Madagascar
Source: BMC Ecol Evol. 2021 Nov 2;21:197. doi: 10.1186/s12862-021-01929-z (PMC8561976; doi:10.1186/s12862-021-01929-z)
Supplement: Supplementary file 1 — Additional file 1. Supplementary text (Text S1–Text S7), figures (Figure S1–Figure S10) and tables (Table S1–Table S10). [file 12862_2021_1929_MOESM1_ESM.pdf]

Additional file 1 for

Impact of model assumptions on demographic inferences: the case study of two sympatric mouse lemurs in northwestern Madagascar

Helena Teixeira[1]*, Jordi Salmona[2], Armando Arredondo[3,4], Beatriz Mourato[3], Sophie Manzi[2], Romule Rakotondravony[5,6], Olivier Mazet[4], Lounès Chikhi[2,3], Julia Metzger[7,8], Ute Radespiel[1]*

[1] Institute of Zoology, University of Veterinary Medicine Hannover, Foundation, Bünteweg 17, Hannover, 30559, Germany

[2] Laboratoire Évolution & Diversité Biologique (EDB UMR 5174), Université de Toulouse Midi-Pyrénées, CNRS, IRD, UPS. 118 route de Narbonne, Bât. 4R1, 31062 Toulouse cedex 9, France

[3] Instituto Gulbenkian de Ciência, Rua da Quinta Grande, 6, P-2780-156 Oeiras, Portugal

[4] Université de Toulouse, Institut National des Sciences Appliquées, Institut de Mathématiques de Toulouse, Toulouse, France

[5] Ecole Doctorale Ecosystèmes Naturels (EDEN), University of Mahajanga, 5 Rue Georges V - Immeuble KAKAL, Mahajanga Be, B.P. 652, Mahajanga 401, Madagascar

[6] Faculté des Sciences, de Technologies et de l'Environnement, University of Mahajanga, 5 Rue Georges V - Immeuble KAKAL, Mahajanga Be, B.P. 652, Mahajanga 401, Madagascar

[7] Institute of Animal Breeding and Genetics, University of Veterinary Medicine Hannover, Foundation, Bünteweg 17p, Hannover, 30559, Germany

[8] Veterinary Functional Genomics, Max Planck Institute for Molecular Genetics, Ihnestrasse 73, 14195 Berlin, Germany

Text S1 – RADseq sequencing library

An average of 8,192,559 ± 4,288,959 (SD; standard deviation) and 11,635,382 ± 11,450,501 Illumina reads per individual were obtained for the 22 *M. murinus* and 56 *M. ravelobensis*, respectively. After applying the quality filters (i.e., removing Illumina barcodes, discarding low quality reads and trimming), an average of 7,084,098 ± 3,728,286 and 7,794,014 ± 3,338,758 reads per individual were retained for *M. murinus* and *M. ravelobensis,* respectively. Of these, an average of 6,660,529 ± 3,521,900 *M. murinus* reads and 7,411,684 ± 3,179,920 *M. ravelobensis* reads per individual were successfully mapped to the reference genome. After PCR duplicate removal, an average of 5,197,520 ± 2,508,055 and 5,818,960 ± 2,293,164 reads per individual were finally retained for the downstream analyses for *M. murinus* and *M. ravelobensis,* respectively (Table S9 and S10).

Text S2 – Whole-genome sequences sequencing library

Whole-genome sequencing and mapping of the single *M. murinus* and the two *M. ravelobensis* individuals resulted in 39,087,552,633 bp (cigar; *M. murinus*), 41,577,088,467 bp (cigar; *M. ravelobensis*, Ravelobe) and 45,840,222,542 bp (cigar; *M. ravelobensis*, Ankomakoma) mapped to the *M. murinus* reference genome (2439.73 Mb), and a corresponding error rate of $2.66*10^{-02}$, $3.64*10^{-02}$ and $3.40*10^{-02}$, respectively.

Text S3 – Relatedness results

The relatedness analyses revealed that two *M. murinus* individuals sampled in Ankomakoma, and two *M. ravelobensis* individuals captured in each sampling site had first degree relatives (i.e., parent/offspring or full sibling) in our dataset (n = 6; data not shown). One of each closely related dyad partners was removed from the dataset and no longer considered in the downstream population genomics analyses. Therefore, the final dataset for the remaining analyses comprised 22 *M. murinus* (7 from Ravelobe and 15 from Ankomakoma) and 56 *M. ravelobensis* (31 from Ravelobe and 25 from Ankomakoma).

Text S4 – Genomic diversity in *M. murinus* and *M. ravelobensis*

Population-genomic diversity in each study site was estimated based on the percentage of polymorphic sites, unbiased expected heterozygosity (He; estimated according to [1]) observed

heterozygosity (Ho) and inbreeding coefficients (F_{IS}). All genetic summary statistics were calculated with ARLEQUIN [2] after conversion of the Variant Call Format (VCF) file from dataset 2 into the ARLEQUIN format using PGDSpider [3].

Despite being represented by a smaller sample, *M. murinus* exhibited consistently higher levels of genomic diversity than *M. ravelobensis* in each forest site (Table S5). The observed heterozygosity ranged between 0.220 (*M. ravelobensis*, Ravelobe) and 0.364 (*M. murinus*, Ravelobe). Both species showed non-significant negative F_{IS} values ranging from -0.069 to -0.203 ($P > 0.05$), suggesting no systematic departures from Hardy-Weinberg Equilibrium (HWE).

The genomic diversity statistics estimated for *M. murinus* and *M. ravelobensis* (Table S5) were lower than previous values estimated with microsatellites datasets [4–6], but comparable to those reported for other primate species based on a RADseq dataset (e.g., Ho = 0.3475 and 0.3148 for *Rhinopithecus roxellana* populations) [7], and even higher than for some other taxa (e.g., [8–10]). A slightly higher observed heterozygosity than expected under HWE coupled with the non-significant but negative population inbreeding coefficients suggest that populations were large enough to retain genetic diversity [5, 11], or that outside individuals were regularly immigrating into the two forest sites.

Text S5 – Impact of the minimum read depth in the *PSMC* analyses

The *PSMC* runs considering different minimum read depth options (–d1 to –d12) resulted in similar demographic curves for *M. ravelobensis* (Ankomakoma; Figure S9). When decreasing the –d option from twelve to one, the genome-wide coverage only decreased from 18.79X to 18.2X, suggesting that the sites considered in the *PSMC* analyses were of good quality. Moreover, the two *M. ravelobensis* sequences suggested a similar demographic history for Ankomakoma and Ravelobe, despite the mean genome-wide coverage differences (18.79 and 17.04, respectively). These results suggest that our *PSMC* inferences are likely not strongly biased by a mean genome-wide coverage below 18X.

Text S6 – List of the ms commands used for establishing the *IICR* figures

<u>For *M. murinus* (Figure 3c)</u>

The *IICR* of *M. murinus* (Figure 3c) was computed considering the following ms command:

ms 2 100000 -T -L -I 84 2 0 0 0 0 0 0 0 0 0 0 0 0 0 0 0 0 0 0 0 0 0 0 0 0 0 0 0 0 0 0 0 0 0 0 0 0 0 0 0 0 0 0 0 0 0 0 0 0 0 0 0 0 0 0 0 0 0 0 0 0 0 0 0 0 0 0 0 0 0 0 0 0 0 0 0 0 0 0 0 0 0 0 0 **1.22401** -eN 0 1.0 -eM **3.6948432453522733** **4.12863** -eN 3.6948432453522733 1.0 -eM **9.925975968381731** **0.928966** -eN 9.925975968381731 1.0 -eM **22.20251298762292** **2.53288** -eN 22.20251298762292 1.0 -eM **30.82745066870904** **86.0061** -eN 30.82745066870904 1.0 -eM **93.26215128503407** **0.281376** -eN 93.26215128503407 1.0

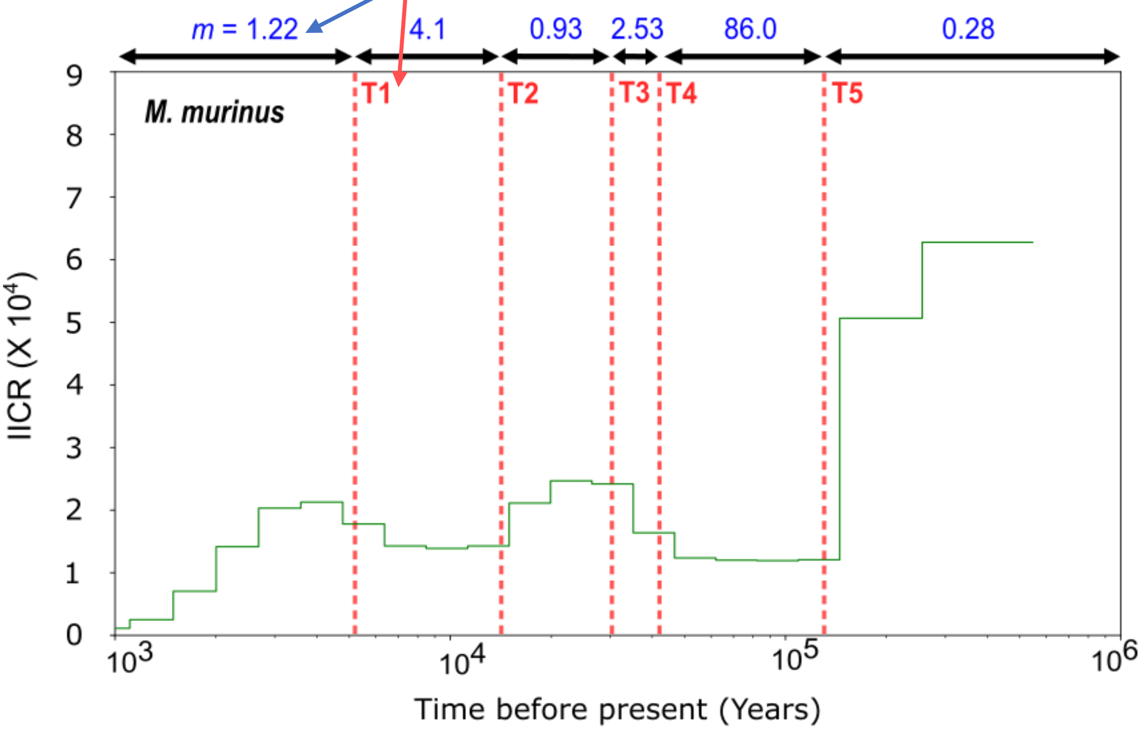

This ms command specifies that *M. murinus* was structured in a n-island model of migration with a constant population size over time and underwent five major changes in population connectivity. The demographic parameters were specified by the following command options:

–I (number of islands) = 84

–eN *t x* (set all subpopulations to size $x * N_0$), where x = 1.0 throughout the time

–eM t *m* (set all elements of the migration matrix to *m*/(npop−1) at time t), where *m* = 1.22; 4.1; 0.93; 2.53; 86.0; 0.28 (backwards in time)

t (specifies the time of a given demographic event in units of $4N_0$ generations). Considering N_0 ~ 138 and a generation time of 2.5 years, the most recent change in population connectivity (T1) took place at 3.695 X $4N_0$ X generation time ~ 5,099 years. If applying the same logic to the remaining *t* values, the changes in population connectivity took place approximately 5.1; 13.7; 30.7; 42.7 and 129.1 kyr.

For *M. ravelobensis* (Figure 3d):

The *IICR* of *M. ravelobensis* was computed considering the following ms command:

ms 2 100000 -T -L -I 61 2 0 0 0 0 0 0 0 0 0 0 0 0 0 0 0 0 0 0 0 0 0 0 0 0 0 0 0 0 0 0 0 0 0 0 0 0 0 0 0 0 0 0 0 0 0 0 0 0 0 0 0 0 0 0 0 0 0 0 0 0 0 0 0 **2.42452** -eN 0 1.0 -eM **1.90869939707149** **0.654211** -eN 1.90869939707149 1.0 -eM **4.8831325593526715** **1.97206** -eN 4.8831325593526715 1.0 -eM **6.566522303502324** **5.38629** -eN 6.566522303502324 1.0 -eM **32.895633924130486** **86.3802** -eN 32.895633924130486 1.0 -eM **82.23483883490435** **9.26377** -eN 82.23483883490435 1.0.

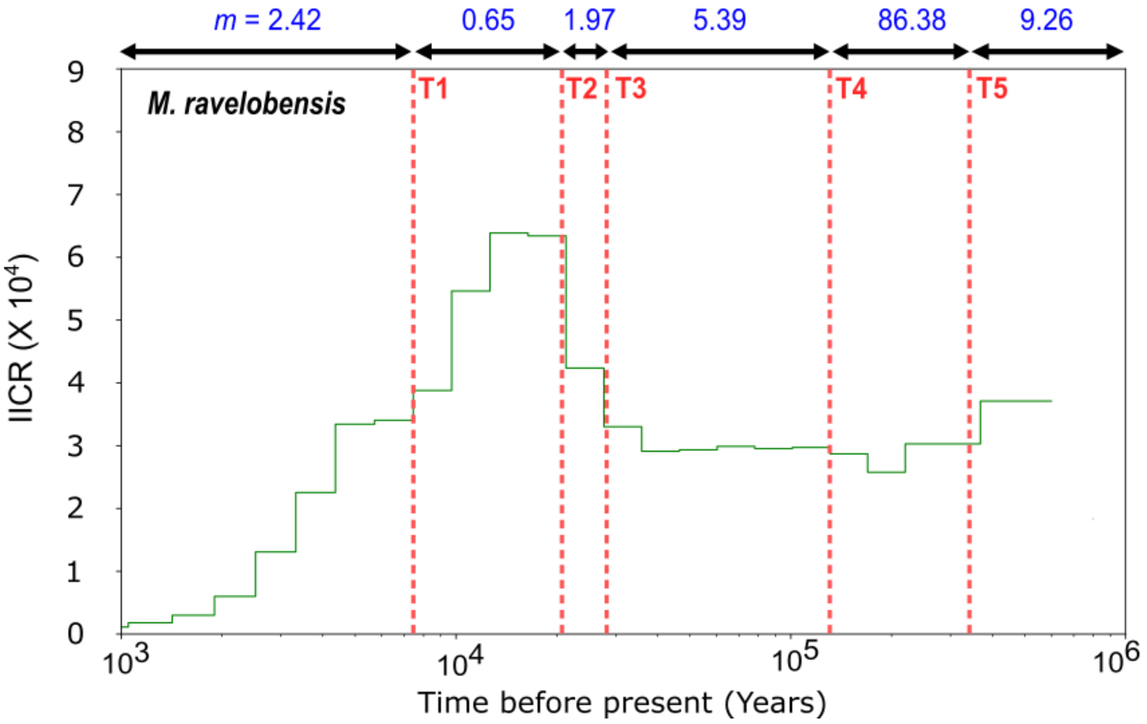

Similarly to *M. murinus*, the ms command specifies that *M. ravelobensis* was structured in a n-island model of migration with a constant population size over the time and underwent five major changes in population connectivity. The demographic parameters were specified by the following command options:

–I (number of islands) = 61

–*m* = 2.42; 0.65; 1.97; 5.39; 86.38; 9.26 (backwards in time)

-*t* = 7.9; 20.1; 27.1; 135.6; 338.9 kyr, when considering N_0 ~ 412 and a generation time of 2.5 years.

Text S7 – Impact of repeat regions in the demographic modelling

To evaluate the impact of repeat regions in our demographic inferences, we reran the *Stairway Plot* for the entire *M. ravelobensis* dataset (n = 55) and the *PSMC* analyses for the three whole-genome sequences without the repeat regions. None of the new results showed substantial deviations from previous results generated using the full dataset and included in the main text (Figure 3a,b). The *PSMC* analyses without the repeat regions (Figure S10a) exhibited a similar demographic trend for *M. murinus* as the analyses using the full dataset (Figure 3a). The *PSMC* dynamics with and without the repeat regions for *M. ravelobensis* were also similar, except for the population bottleneck that occurred during the African Humid Period. This event was no longer detected when considering the dataset without repeat regions. Such differences are likely related to the substantial reduction on the number of informative sites after removal of the repeat regions (approximately 52%). Since the *Stairway plot* results without the repeat regions (Figure S10b) revealed an identical curve to the one inferred using all the sites (Figure 3b), we conclude that the demographic inferences for our study species were not impacted by the inclusion of repeat regions.

a

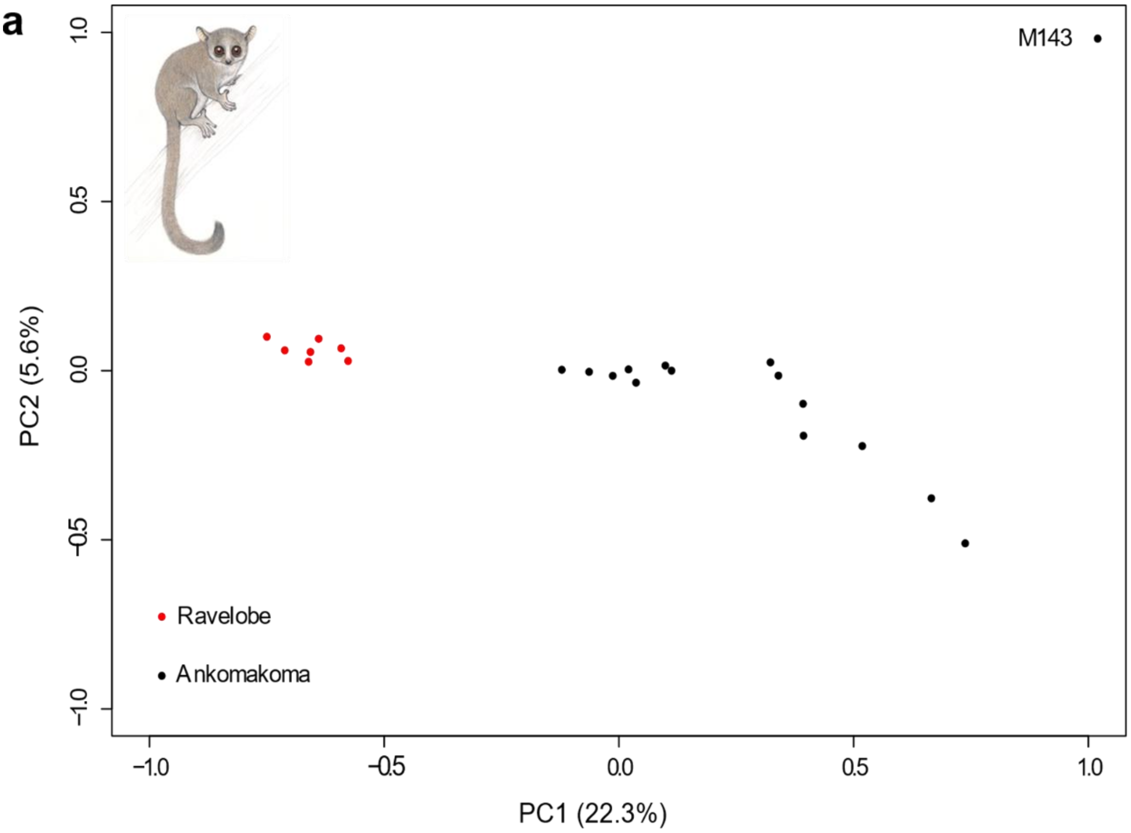
M143
Ravelobe
Ankomakoma
PC2 (5.6%)
PC1 (22.3%)
1.0
0.5
0.0
−0.5
−1.0
−1.0
−0.5
0.0
0.5
1.0

b

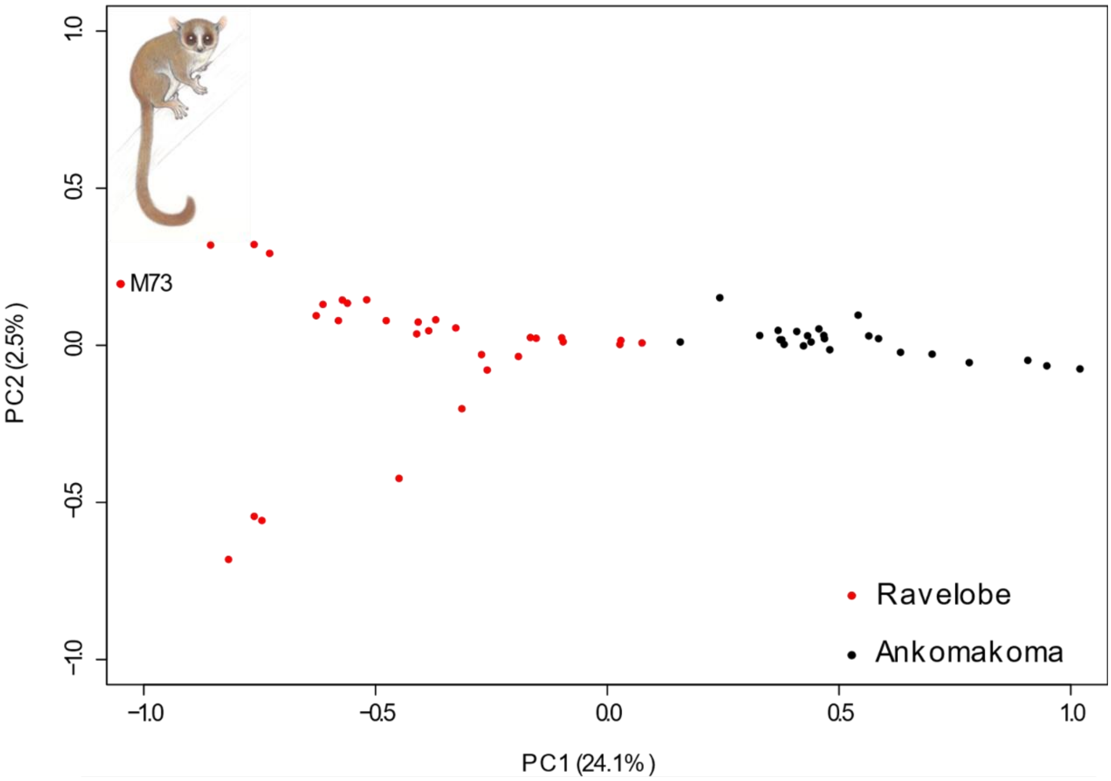
M73
Ravelobe
Ankomakoma
PC2 (2.5%)
PC1 (24.1%)
1.0
0.5
0.0
−0.5
−1.0
−1.0
−0.5
0.0
0.5
1.0

Figure S1. Principal Component Analyses plots based on genotype likelihoods in **a** *M. murinus* (N = 22) and **b** *M. ravelobensis* (N = 56) using the same datasets as for the clustering analyses. The axis labels show the variation explained by the first two principal components (PC1 and PC2). Results suggest weak genetic structure among the two sampling sites for both species. Although the PCA analyses revealed one genetically distinct *M. murinus* individual (M143), this animal was kept in our analyses, because no differences were observed in the clustering analyses (Figure 2 and Figure S2), the inbreeding coefficient suggested no departure from Hardy-Weinberg Equilibrium (HWE; Table S6), and the sample showed a good individual mean coverage depth (Table S2). In contrast, *M. ravelobensis* individual M73 was excluded from our subsequent analyses because it exhibited an $F = -0.476$ (Table S6), strongly deviating from HWE, which may impact downstream analyses. Animal illustrations copyright 2013 Stephen D. Nash / IUCN SSC Primate Specialist Group. Used with permission.

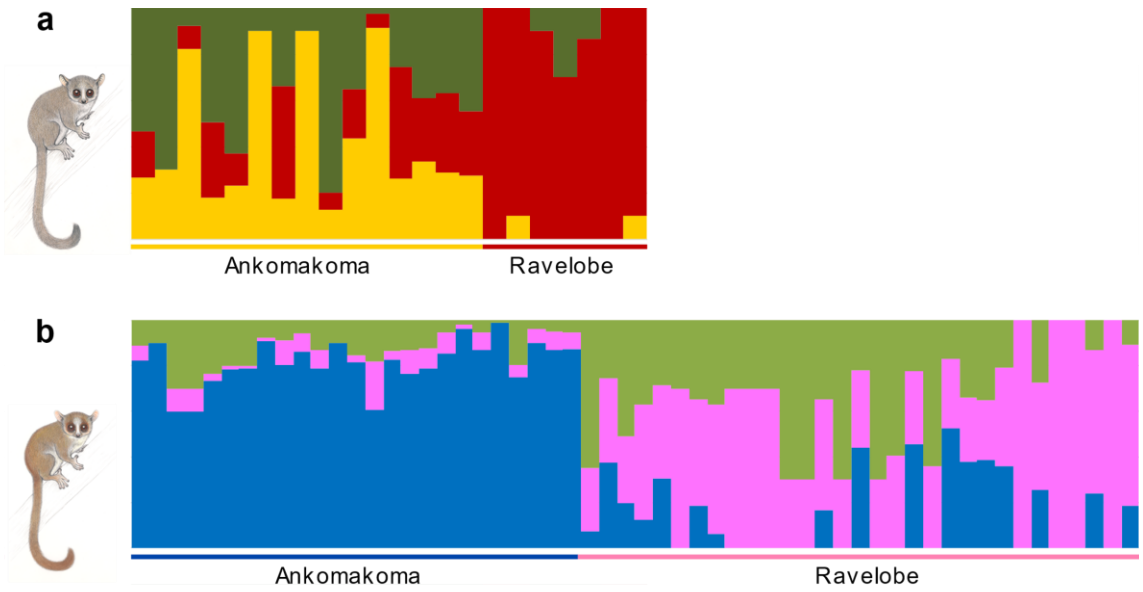

Figure S2. Results of a population genomic structure analysis of the two mouse lemur species. **a** Cluster assignment of 22 *M. murinus* individuals and **b** Cluster assignment of 56 *M. ravelobensis* individuals to three genetic clusters under K = 3 using dataset 1. Each single vertical bar represents one individual and each color a distinct genetic cluster. Samples are sorted according to sampling site and respective latitude. Animal illustrations copyright 2013 Stephen D. Nash / IUCN SSC Primate Specialist Group. Used with permission.

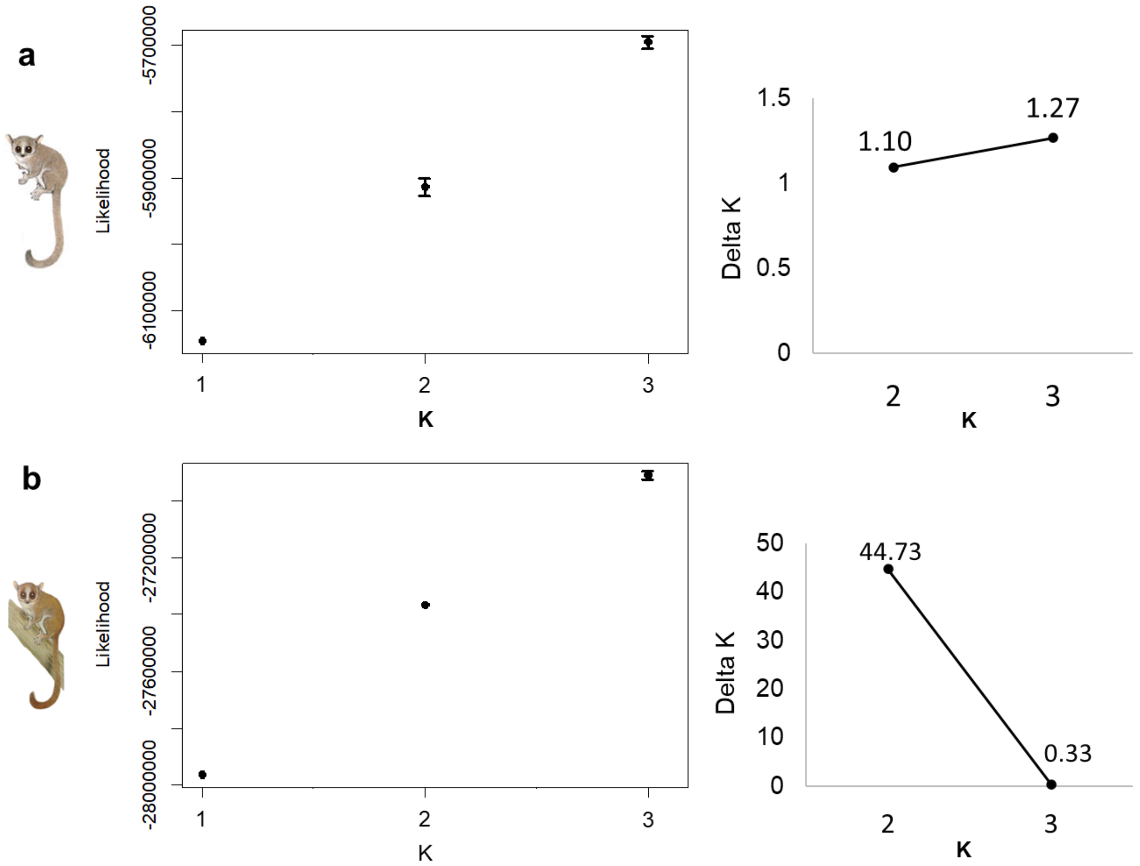

Figure S3. Number of clusters inferred by NGSadmix. **a** Likelihood results and delta K estimation suggest that the best K value for *M. murinus* is K = 3. **b** Likelihood results and delta K estimation suggest that the best K value for *M. ravelobensis* is K = 2. Delta K estimations followed the method of [12] over 10 replicate NGSadmix runs for each K value. Animal illustrations copyright 2013 Stephen D. Nash / IUCN SSC Primate Specialist Group. Used with permission.

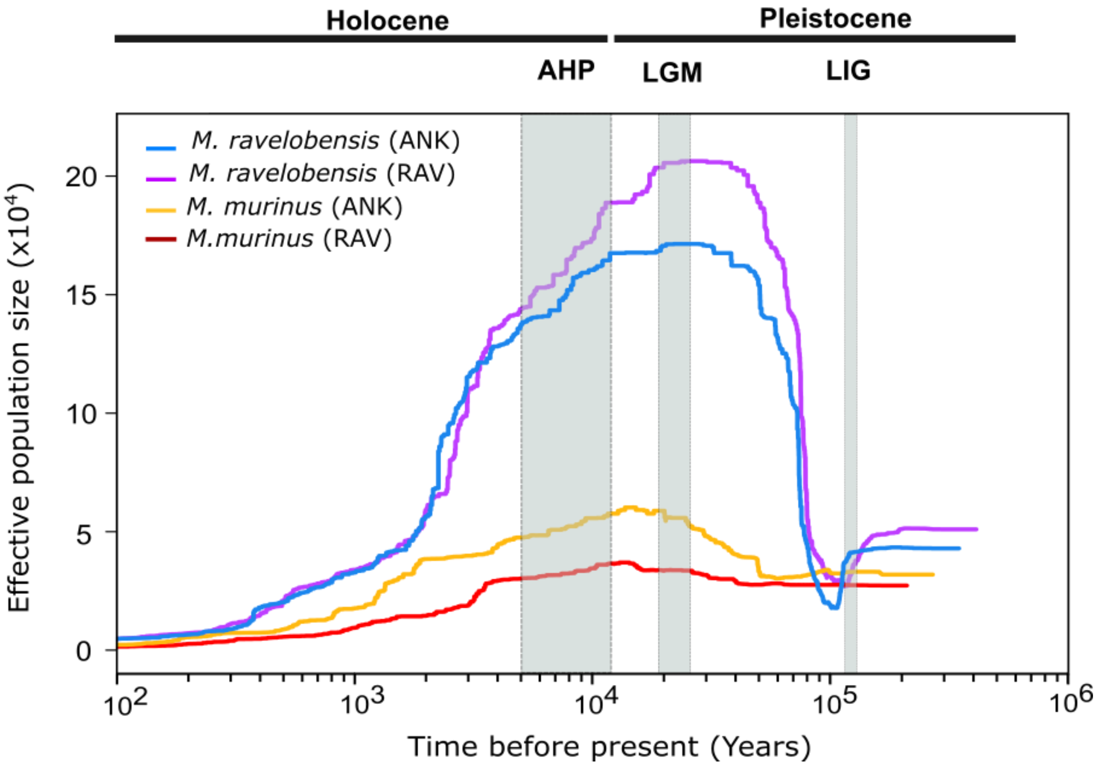

Figure S4. Demographic history inferred by the Stairway Plot method based on the entire dataset (*M. murinus*, Ravelobe = 7; *M. murinus*, Ankomakoma = 15; *M. ravelobensis*, Ravelobe = 30; *M. ravelobensis*, Ankomakoma = 25). The analyses were performed considering 2.5 years as generation time and 1.2 × 10^{-8} as mutation rate. The grey vertical bars identify three well-pronounced climatic events that took place in Africa: LIG (Last Interglacial), LGM (Last Glacial Maximum) and AHP (African Humid Period). RAV = Ravelobe; ANK = Ankomakoma

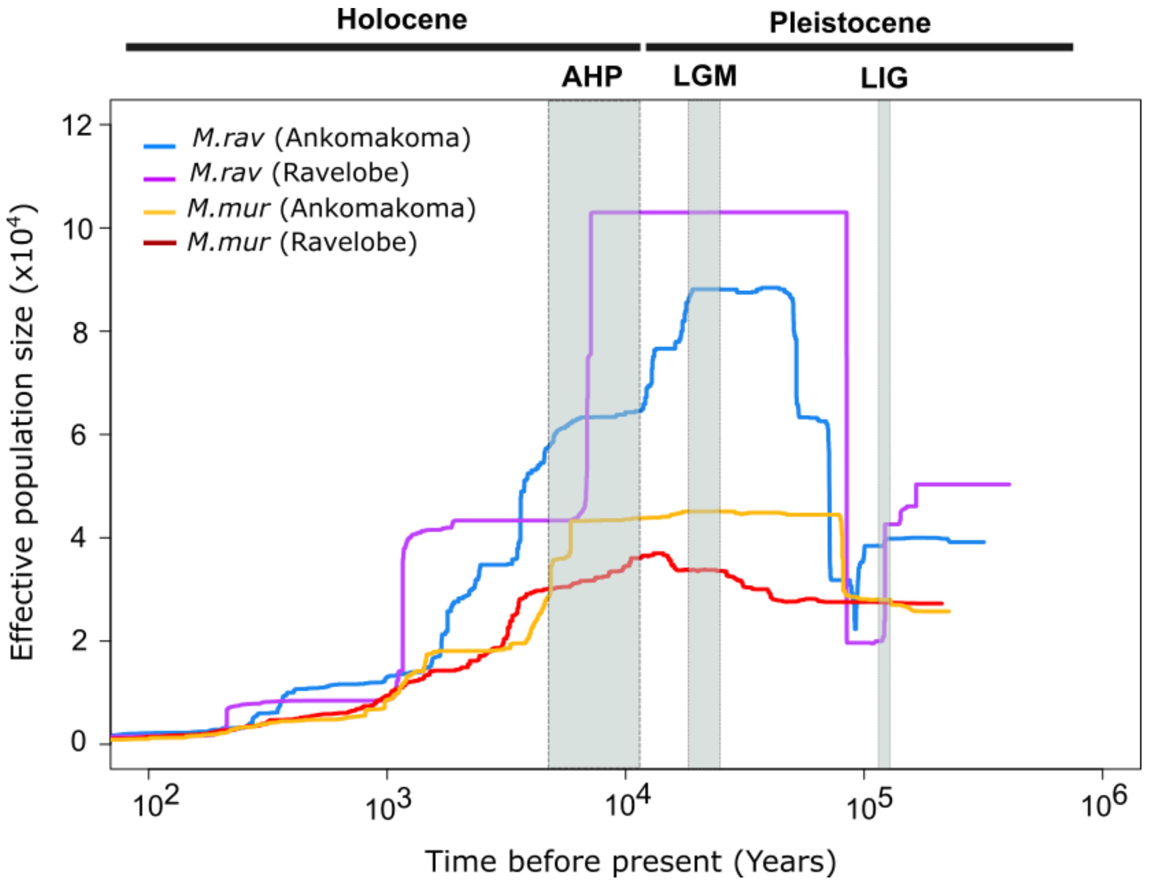

Figure S5. Reconstruction of the demographic history of *M. murinus* (*M. mur*) and *M. ravelobensis* (*M. rav*) using the *Stairway Plot* method, considering the same sample size for all populations (n = 7). Individuals were selected randomly using R. All analyses were performed considering 2.5 years as generation time and 1.2×10^{-8} as mutation rate. The grey vertical bars identify three well-pronounced climatic events that took place in Africa: LIG (Last Interglacial), LGM (Last Glacial Maximum) and AHP (African Humid Period).

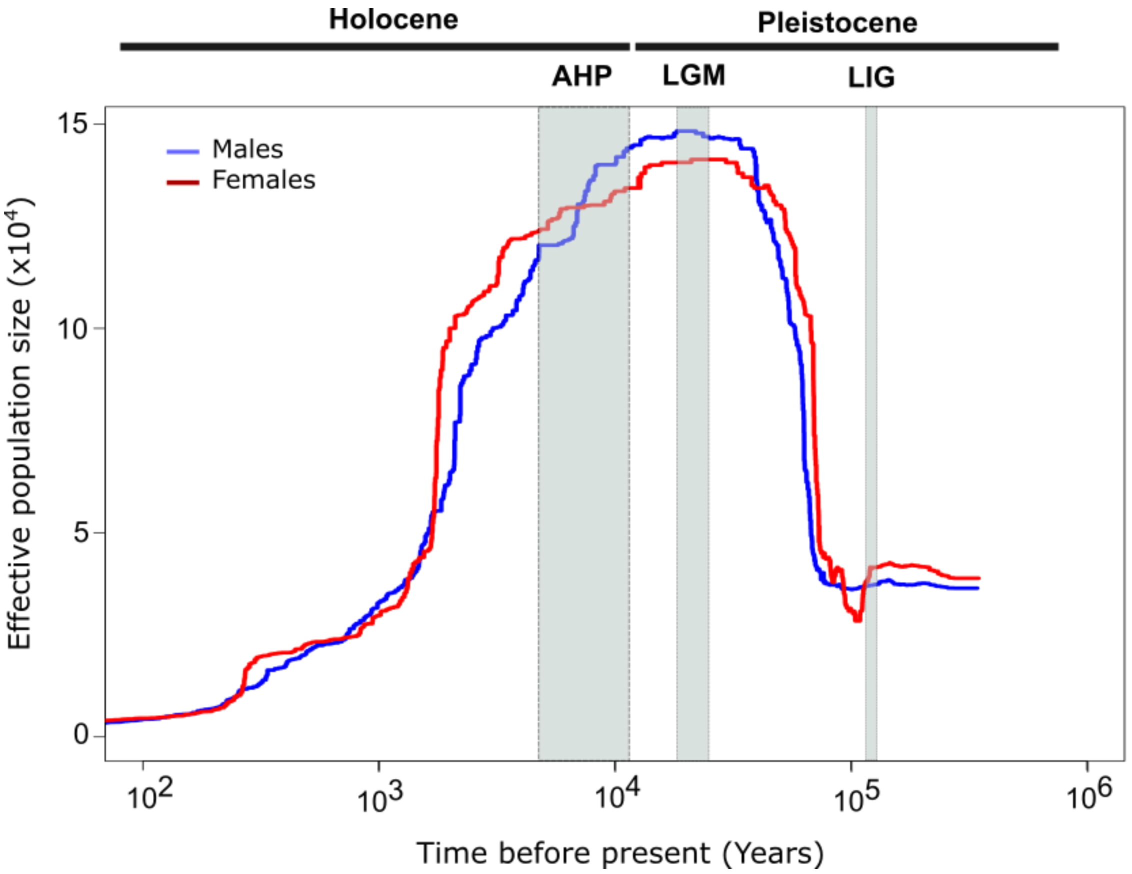

Figure S6. Reconstruction of the demographic history of *M. ravelobensis* using the *Stairway Plot* method, considering males and females separately. The analyses were performed considering all *M. ravelobensis* females available in our dataset (n = 22) and an equal number of males. The males were selected randomly using R. All analyses were performed considering 2.5 years as generation time and 1.2×10^{-8} as mutation rate. The grey vertical bars identify three well-pronounced climatic events that took place in Africa: LIG (Last Interglacial), LGM (Last Glacial Maximum) and AHP (African Humid Period).

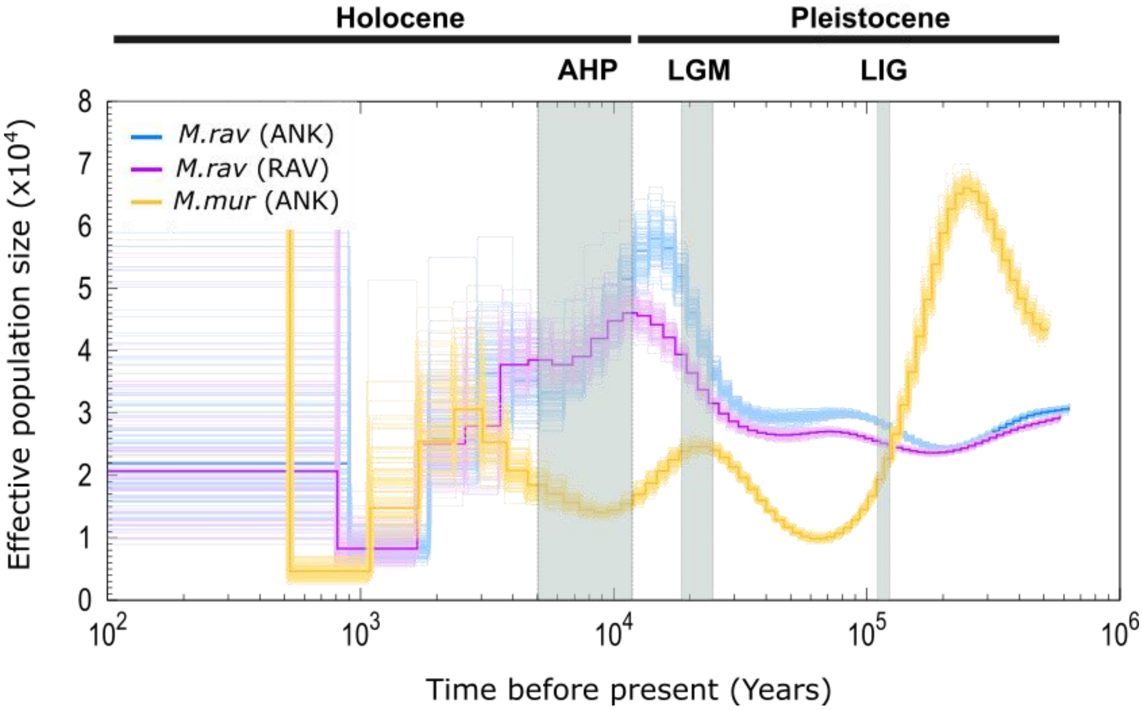

Figure S7. Demographic history inferred by the *PSMC* method using "64*1" free atomic time intervals. The thick lines represent the inferred mean trajectories for three populations, and each light line represents one of 100 subsampled bootstrap replicates for each individual. The humps observed in *PSMC* plot of the two mouse lemur species during the last 2 – 5 kyr (Figure 3a) are no longer observed when considering "64*1" free atomic time intervals, suggesting that those humps are an artefact. The analyses were performed considering 2.5 years as generation time and 1.2×10^{-8} as mutation rate. The grey vertical bars identify three well-pronounced climatic events that took place in Africa: LIG (Last Interglacial), LGM (Last Glacial Maximum) and AHP (African Humid Period). M. rav = *M. ravelobensis*; M. mur = *M. murinus*; RAV = Ravelobe; ANK = Ankomakoma.

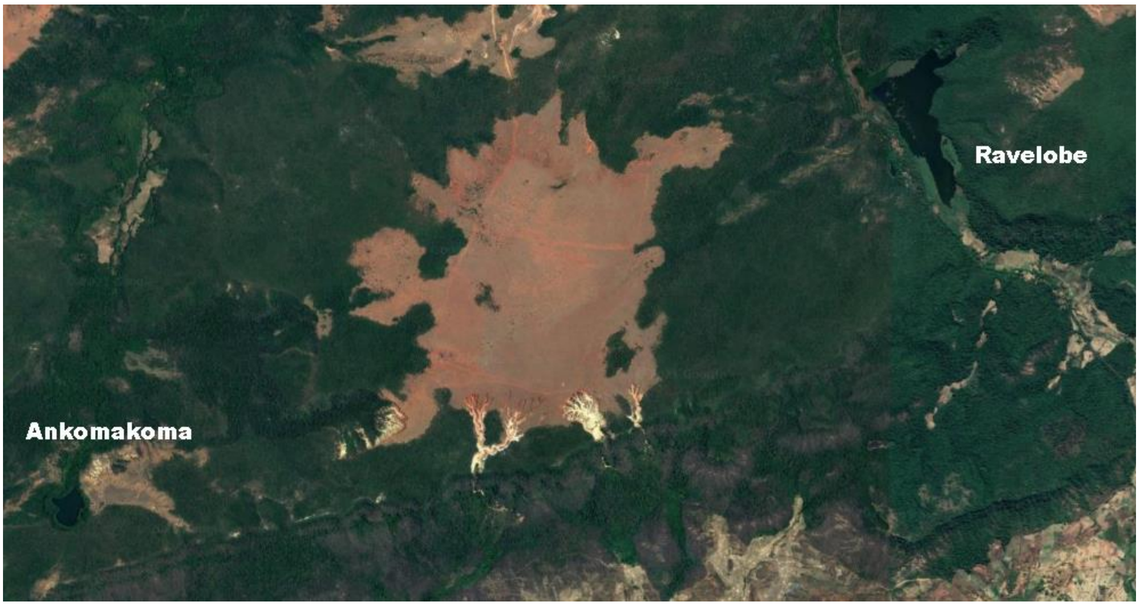

Figure S8. Satellite view of our study sites. Although Ankomakoma and Ravelobe are part of a mosaic landscape, the two sites are still connected via forest corridors that surrounded the savannah. Image was taken from *Google Earth* [13].

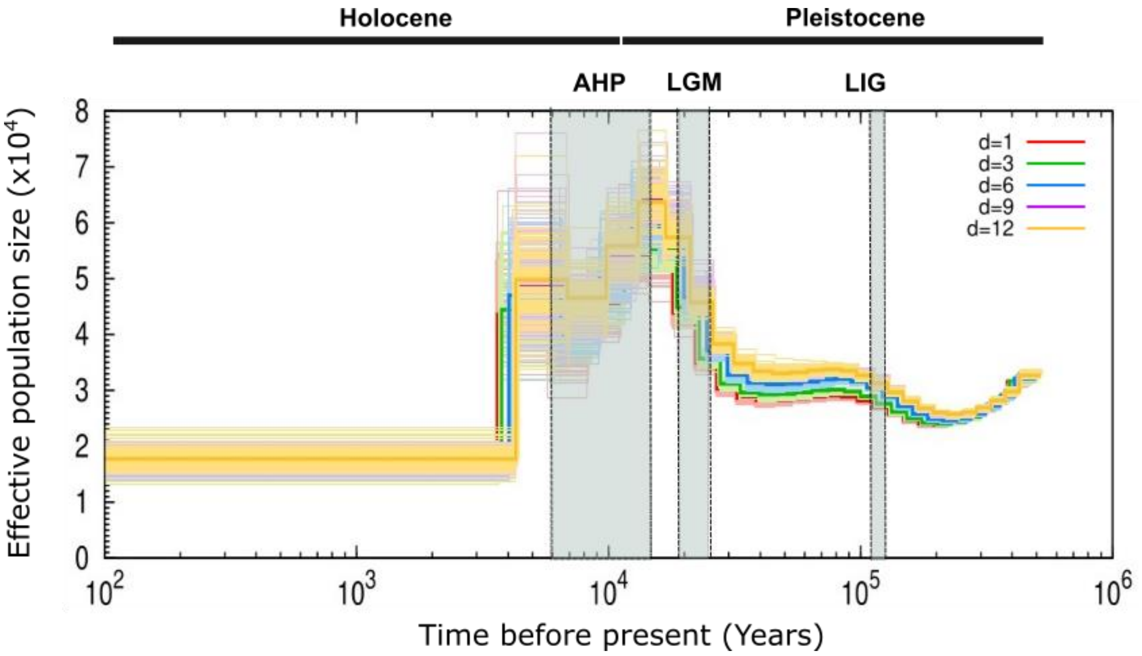

Figure S9. Impact of varying the minimum read depth (–d) on the *PSMC* analyses for *M. ravelobensis* (Ankomakoma). The demographic dynamics of *M. ravelobensis* using different minimum read depth options (–d1, –d3, –d6, –d9, –d12) resulted in similar demographic curves, suggesting that the sites considered in the *PSMC* analyses were of good quality. The analyses were performed considering 2.5 years as generation time and 1.2×10^{-8} as mutation rate. The grey vertical bars identify three well-pronounced climatic events that took place in Africa: LIG (Last Interglacial), LGM (Last Glacial Maximum) and AHP (African Humid Period).

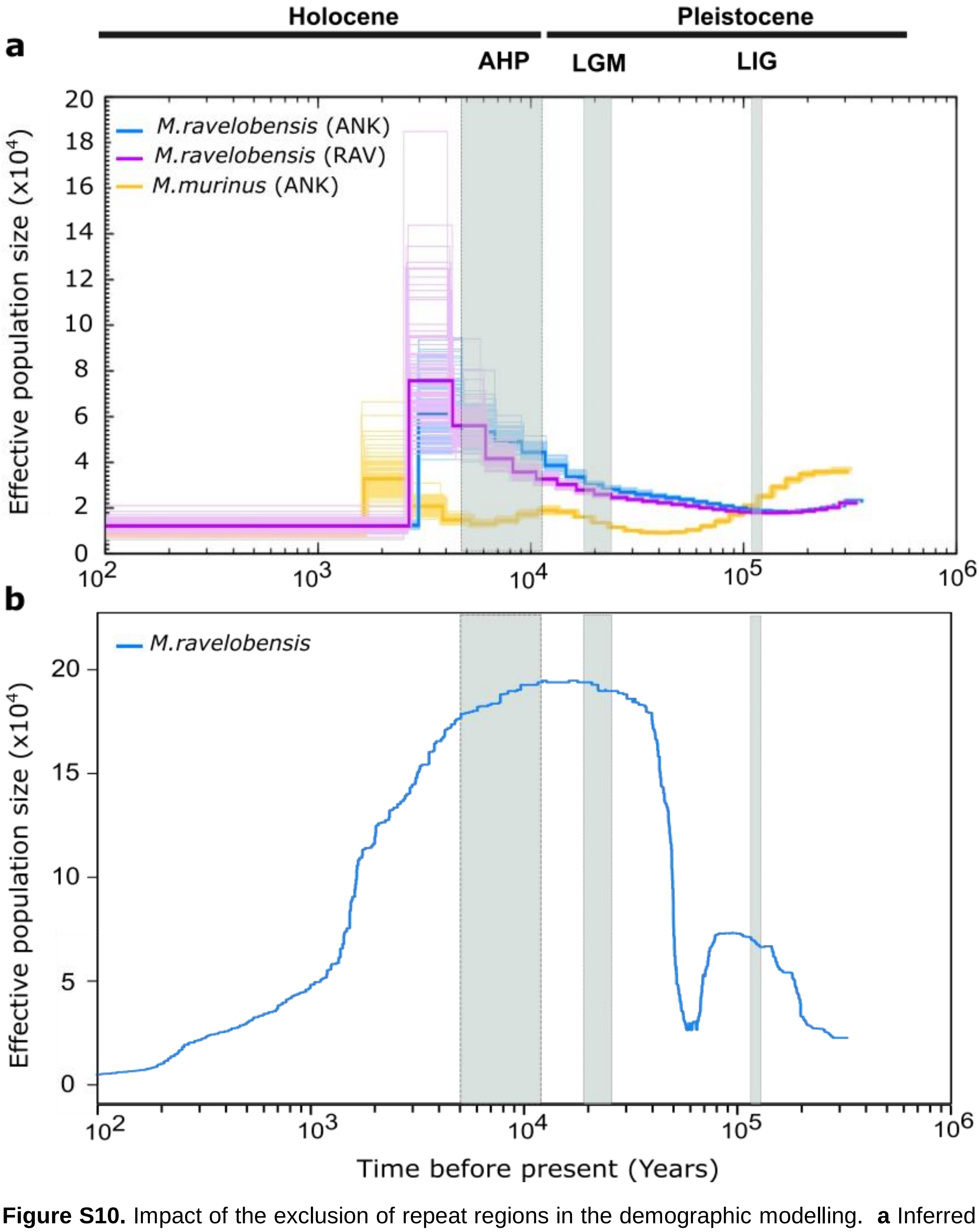

Figure S10. Impact of the exclusion of repeat regions in the demographic modelling. **a** Inferred trajectories for *M. murinus* and *M. ravelobensis* after the exclusion of the repeat regions using the *PSMC*. The results are identical to the ones generated using the full dataset, except for the population bottleneck that occurred during the African Humid Period for the *M. ravelobensis*. These results suggest that larger datasets are essential to correctly infer recent demographic dynamics with this method. **b** Inferred trajectory for *M. ravelobensis* (n = 55) after the exclusion of the repeat regions using the *Stairway Plot* method. The results are identical to the ones generated using the full dataset. The grey vertical bars identify three well-pronounced climatic events that took place in Africa: LIG (Last Interglacial), LGM (Last Glacial Maximum) and AHP (African Humid Period).

Table S1. Elevation range in which each species was trapped along each transect in both study sites. M.rav: *M. ravelobensis*; M.mur: *M. murinus*; a.s.l.: above sea level. See Figure 1b,c for visual representation of the transects.

Study site	Transect	Species occurence	Occurence of *M. ravelobensis* (m a.s.l.)	Occurence of *M. murinus* (m a.s.l.)
Ravelobe	T1	M.rav	101 - 159	_
	T2	M.rav	109 - 114	_
	T3	M.rav	88 - 90	_
	T4	M.rav; M.mur	119 - 140	133 - 161
Ankomakoma	T1	M.rav	108 - 129	_
	T2	M.rav; M.mur	105 - 132	112 - 125
	T3	M.mur	_	106 - 153
	T4	M.rav	137 - 162	_

Table S2. Mean depth for the *M. murinus* individuals used for the genomic analyses (n = 22). The mean depth per individual ranged between 13.82 and 42.18. N sites = number of sites per individual used to estimate the mean depth per individual with VCFtools.

Study site	Sample ID	N sites	mean depth
Ravelobe	M53	105233	15.23
Ravelobe	M54	121972	29.72
Ravelobe	M59	116579	17.76
Ravelobe	M91	101100	14.96
Ravelobe	M92	121084	22.52
Ravelobe	M93	95655	14.72
Ravelobe	F94	59066	13.82
Ankomakoma	F104	119616	19.40
Ankomakoma	F106	122023	32.01
Ankomakoma	F107	122037	42.18
Ankomakoma	F108	111094	15.96
Ankomakoma	F118	103530	14.97
Ankomakoma	F145	122037	38.78
Ankomakoma	F148	121883	26.38
Ankomakoma	M109	122016	38.23
Ankomakoma	M110	107273	15.45
Ankomakoma	M112	122043	36.63
Ankomakoma	M115	104094	15.06
Ankomakoma	M116	97809	14.59
Ankomakoma	M143	121947	40.34
Ankomakoma	M147	122006	32.40
Ankomakoma	M153	120117	20.87

Table S3. Mean depth for the *M. ravelobensis* individuals used for the genomic analyses (n = 55). The mean depth per individual ranged between 13.96 and 47.88. N sites = number of sites per individual used to estimate the mean depth per individual with VCFtools.

Study site	Sample ID	N sites	Mean depth
Ravelobe	F20	241353	22.92
Ravelobe	F21	220745	15.60
Ravelobe	F22	236833	18.26
Ravelobe	F25	241846	27.66
Ravelobe	F30	241451	23.63
Ravelobe	F41	241856	25.41
Ravelobe	F62	217447	19.74
Ravelobe	F77	211980	15.32
Ravelobe	F80	211035	15.38
Ravelobe	F82	242039	30.03
Ravelobe	F83	240608	21.48
Ravelobe	F87	158148	13.96
Ravelobe	F88	221094	15.63
Ravelobe	M18	241860	26.96
Ravelobe	M19	241945	26.68
Ravelobe	M29	234896	17.71
Ravelobe	M42	226819	16.36
Ravelobe	M48	232774	17.22
Ravelobe	M52	218556	15.46
Ravelobe	M55	239865	20.24
Ravelobe	M56	234718	17.44
Ravelobe	M57	241744	25.52
Ravelobe	M58	241827	25.96
Ravelobe	M60	239142	20.20
Ravelobe	M67	241688	25.04
Ravelobe	M68	239012	21.49
Ravelobe	M79	241424	23.45
Ravelobe	M81	241879	27.19
Ravelobe	M90	206033	14.55
Ravelobe	M95	241845	26.99
Ankomakoma	F119	242087	38.59
Ankomakoma	F126	242083	33.95
Ankomakoma	F129	242072	46.77
Ankomakoma	F132	242080	42.47
Ankomakoma	F157	242026	30.80
Ankomakoma	F165	242080	35.65
Ankomakoma	F166	242057	35.96
Ankomakoma	F169	242069	36.66
Ankomakoma	F98	194516	14.29
Ankomakoma	F99	240296	21.32

Ankomakoma	M101	242113	40.43
Ankomakoma	M102	242079	37.27
Ankomakoma	M120	241952	28.99
Ankomakoma	M121	242081	38.56
Ankomakoma	M127	242108	47.88
Ankomakoma	M130	241596	24.97
Ankomakoma	M135	242100	44.92
Ankomakoma	M138	242079	45.60
Ankomakoma	M140	241188	25.73
Ankomakoma	M141	242060	44.51
Ankomakoma	M154	242007	30.78
Ankomakoma	M158	242037	33.51
Ankomakoma	M159	242063	39.08
Ankomakoma	M161	242063	43.70
Ankomakoma	M163	241718	25.66

Table S4. Summary of the demographic dynamics reconstructions for *M. ravelobensis* and *M. murinus* assuming population panmixia (*PSMC* and *Stairway Plot*) or population structure (*IICR*-simulations). The demographic dynamics that were in line with the available paleoenvironmental and the resulting demographic hypothesis are marked with a green check, while the dynamics that were not in concordance with the respective hypothesis are marked with a not applicable icon. The demographic dynamics of both mouse lemur species were better explained by a mix of population size and connectivity changes. These results suggest that population structure played an important role in the demographic dynamics of *M. ravelobensis* and *M. murinus*. NA = Hypothesis is not applicable to this study species.

		M. ravelobensis		***M. murinus***	
**Climatic event**	**Demographic hypothesis**	**Panmixia**	**Structure**	**Panmixia**	**Structure**
Late Pleistocene	*M. murinus*: founder effect during its colonization of NW Madagascar	NA	NA	✓	NA
LGM	*M. ravelobensis*: bottleneck and/or reduced connectivity	⊘	✓	NA	NA
AHP	*M. ravelobensis* & *M. murinus*: population expansion and/or higher connectivity	⊘	⊘	⊘	✓
AHP termination	*M. ravelobensis* & *M. murinus*: bottleneck and/or reduced connectivity	✓	✓	✓	✓

Table S5. Genomic diversity summary statistics for each *M. murinus* and *M. ravelobensis* study site using dataset 2. Rav = Ravelobe; Ank = Ankomakoma; n = Sample size (where M = number of males and F = number of females); % Poly = % Polymorphic sites; H_e = unbiased expected heterozygosity (estimated according to [6]); H_o = observed heterozygosity; F_{IS} = population inbreeding coefficient (n.s.; p > 0.05).

Species	Pop	n	% Poly	H_e	H_o	F_{IS}
M. murinus	Rav	7 (7M)	11.5	0.323 (± 0.156)	0.364 (± 0.256)	-0.203
	Ank	15 (8M/7F)	22.3	0.266 (± 0.166)	0.288 (± 0.231)	-0.122
M. ravelobensis	Rav	30 (17M/13F)	27.0	0.205 (± 0.164)	0.220 (± 0.206)	-0.101
	Ank	25 (16M/9F)	44.0	0.218 (± 0.163)	0.230 (± 0.199)	-0.069

Table S6. Individual inbreeding coefficients (*F*) estimated for the *M. ravelobensis* dataset (n = 56). *F* = 0 reflects random mating (HWE) and *F* = 1 the absence of heterozygous genotypes or a totally inbred sample [14]. One individual (M73) showed a highly negative *F* value, suggesting a deviation from HWE and it was therefore removed from our dataset.

Site	Sample ID	Inbreeding coefficient (*F*)
Ravelobe	F20	0.071
Ravelobe	F21	0.014
Ravelobe	F22	0.007
Ravelobe	F25	0.084
Ravelobe	F30	0.090
Ravelobe	F41	0.097
Ravelobe	F62	-0.002
Ravelobe	F77	0.087
Ravelobe	F80	0.033
Ravelobe	F82	0.031
Ravelobe	F83	0.088
Ravelobe	F87	0.046
Ravelobe	F88	0.091
Ravelobe	M18	0.091
Ravelobe	M19	0.089
Ravelobe	M29	0.073
Ravelobe	M42	0.011
Ravelobe	M48	0.045
Ravelobe	M52	0.038
Ravelobe	M55	0.075
Ravelobe	M56	0.090
Ravelobe	M57	0.078
Ravelobe	M58	-0.003
Ravelobe	M60	0.022
Ravelobe	M67	0.033
Ravelobe	M68	0.014
**Ravelobe**	**M73**	**-0.476**
Ravelobe	M79	0.069
Ravelobe	M81	0.041
Ravelobe	M90	0.048
Ravelobe	M95	0.069
Ankomakoma	F119	-0.007
Ankomakoma	F126	0.004
Ankomakoma	F129	0.036
Ankomakoma	F132	0.046
Ankomakoma	F157	0.023
Ankomakoma	F165	0.064
Ankomakoma	F166	0.052
Ankomakoma	F169	0.033
Ankomakoma	F98	0.066

Ankomakoma	F99	0.063
Ankomakoma	M101	0.039
Ankomakoma	M102	0.001
Ankomakoma	M120	0.045
Ankomakoma	M121	0.008
Ankomakoma	M127	0.032
Ankomakoma	M130	0.025
Ankomakoma	M135	0.040
Ankomakoma	M138	-0.152
Ankomakoma	M140	0.006
Ankomakoma	M141	0.069
Ankomakoma	M154	0.017
Ankomakoma	M158	0.045
Ankomakoma	M159	0.022
Ankomakoma	M161	0.074
Ankomakoma	M163	0.029

Table S7. Individual inbreeding coefficients (*F*) estimated for the *M. murinus* dataset (n = 22). *F* = 0 reflects random mating (HWE) and *F* = 1 the absence of heterozygous genotypes or a totally inbred sample [14]. No significant deviations from HWE were found in our dataset and all individuals were kept for downstream analyses.

Site	Sample ID	Inbreeding coefficient (*F*)
Ravelobe	M54	-0.070
Ravelobe	M59	-0.021
Ravelobe	M91	-0.006
Ravelobe	M92	-0.012
Ravelobe	M93	0.020
Ravelobe	F94	-0.004
Ankomakoma	F104	0.007
Ankomakoma	F106	-0.027
Ankomakoma	F107	-0.003
Ankomakoma	F108	0.035
Ankomakoma	F118	-0.006
Ankomakoma	F145	-0.008
Ankomakoma	F148	-0.019
Ankomakoma	M109	0.026
Ankomakoma	M110	0.020
Ankomakoma	M112	0.007
Ankomakoma	M115	0.035
Ankomakoma	M116	0.044
Ankomakoma	M143	0.249
Ankomakoma	M147	0.030
Ankomakoma	M153	0.018
Ankomakoma	M53	0.021

Table S8. List of individuals used for the different genomic analyses for each species and respective sampling locations. All individuals (without close relatives) were considered for the clustering (NGSadmix and PCA) and inbreeding analyses. The individual M73 from *M. ravelobensis* (Ravelobe) was not considered in the downstream analyses because it showed departure from HWE. *Stairway Plot* analyses were performed using the entire datasets and with a subset of the samples (n = 7) to control for differences in population size. Finally, the *PSMC* analyses were performed considering one *M. murinus* sampled in Ankomakoma and one *M. ravelobensis* sampled in each study site.

Species	Site	Sample ID	Latitude (°N)	Longitude (°E)	Sex	NGSadmix/ PCA	*Stairway Plot* all	*Stairway Plot* (n = 7)	*PSMC*
M. murinus	Ankomakoma	F104	-16.344838	46.738467	F	X	X	X	
M. murinus	Ankomakoma	F106	-16.346087	46.739956	F	X	X		
M. murinus	Ankomakoma	F107	-16.346319	46.74069	F	X	X	X	
M. murinus	Ankomakoma	F108	-16.343119	46.741413	F	X	X		
M. murinus	Ankomakoma	F118	-16.346289	46.740222	F	X	X		
M. murinus	Ankomakoma	F145	-16.346855	46.744649	F	X	X		X
M. murinus	Ankomakoma	F148	-16.345054	46.7429	F	X	X	X	
M. murinus	Ankomakoma	M109	-16.34321	46.741629	M	X	X		
M. murinus	Ankomakoma	M110	-16.343939	46.742323	M	X	X	X	
M. murinus	Ankomakoma	M112	-16.346244	46.744077	M	X	X		
M. murinus	Ankomakoma	M115	-16.346319	46.74069	M	X	X		
M. murinus	Ankomakoma	M116	-16.346319	46.74069	M	X	X	X	
M. murinus	Ankomakoma	M143	-16.346839	46.745813	M	X	X		
M. murinus	Ankomakoma	M147	-16.345566	46.743299	M	X	X	X	
M. murinus	Ankomakoma	M153	-16.343819	46.742186	M	X	X	X	
M. murinus	Ravelobe	M53	-16.306787	46.826026	M	X	X	X	
M. murinus	Ravelobe	M54	-16.307775	46.825124	M	X	X	X	
M. murinus	Ravelobe	M59	-16.307775	46.825124	M	X	X	X	
M. murinus	Ravelobe	M91	-16.309582	46.823081	M	X	X	X	
M. murinus	Ravelobe	M92	-16.308552	46.82424	M	X	X	X	

M. murinus	Ravelobe	M93	-16.30813	46.824643	M	X	X	X	
M. murinus	Ravelobe	F94	-16.306257	46.82613	F	X	X	X	
M. ravelobensis	Ankomakoma	F119	-16.345218	46.738692	F	X	X	X	
M. ravelobensis	Ankomakoma	F126	-16.34814	46.732226	F	X	X		
M. ravelobensis	Ankomakoma	F129	-16.348432	46.733121	F	X	X		
M. ravelobensis	Ankomakoma	F132	-16.348398	46.733852	F	X	X		
M. ravelobensis	Ankomakoma	F157	-16.348665	46.734507	F	X	X		X
M. ravelobensis	Ankomakoma	F165	-16.333502	46.735526	F	X	X		
M. ravelobensis	Ankomakoma	F166	-16.332714	46.735417	F	X	X		
M. ravelobensis	Ankomakoma	F169	-16.330171	46.73505	F	X	X		
M. ravelobensis	Ankomakoma	F98	-16.333198	46.735454	F	X	X		
M. ravelobensis	Ankomakoma	F99	-16.329861	46.735221	M	X	X	X	
M. ravelobensis	Ankomakoma	M101	-16.343743	46.737262	M	X	X	X	
M. ravelobensis	Ankomakoma	M102	-16.344501	46.738275	M	X	X		
M. ravelobensis	Ankomakoma	M120	-16.344292	46.737947	M	X	X	X	
M. ravelobensis	Ankomakoma	M121	-16.343307	46.736867	M	X	X	X	
M. ravelobensis	Ankomakoma	M127	-16.348243	46.732361	M	X	X		
M. ravelobensis	Ankomakoma	M130	-16.348552	46.733402	M	X	X		
M. ravelobensis	Ankomakoma	M135	-16.335652	46.737288	M	X	X	X	
M. ravelobensis	Ankomakoma	M138	-16.331646	46.735075	M	X	X		
M. ravelobensis	Ankomakoma	M140	-16.332035	46.735127	M	X	X		
M. ravelobensis	Ankomakoma	M141	-16.329984	46.735127	M	X	X		
M. ravelobensis	Ankomakoma	M154	-16.34896	46.735655	M	X	X		
M. ravelobensis	Ankomakoma	M158	-16.347931	46.731252	M	X	X		
M. ravelobensis	Ankomakoma	M159	-16.347938	46.73081	M	X	X		
M. ravelobensis	Ankomakoma	M161	-16.347608	46.729031	M	X	X	X	
M. ravelobensis	Ankomakoma	M163	-16.335266	46.736924	M	X	X		
M. ravelobensis	Ravelobe	M18	-16.308878	46.80416	M	X	X	X	

M. ravelobensis	Ravelobe	M19	-16.307487	46.806598	M	X	X	X	
M. ravelobensis	Ravelobe	M29	-16.302069	46.81621	M	X	X		
M. ravelobensis	Ravelobe	M42	-16.308663	46.819754	M	X	X		
M. ravelobensis	Ravelobe	M48	-16.3146	46.822572	M	X	X		
M. ravelobensis	Ravelobe	M52	-16.309204	46.823946	M	X	X	X	
M. ravelobensis	Ravelobe	M55	-16.306982	46.807239	M	X	X		
M. ravelobensis	Ravelobe	M56	-16.307058	46.809144	M	X	X		
M. ravelobensis	Ravelobe	M57	-16.312068	46.82317	M	X	X		
M. ravelobensis	Ravelobe	M58	-16.308708	46.824182	M	X	X		
M. ravelobensis	Ravelobe	M60	-16.308898	46.824186	M	X	X		
M. ravelobensis	Ravelobe	M67	-16.300921	46.821531	M	X	X		
M. ravelobensis	Ravelobe	M68	-16.301686	46.820723	M	X	X	X	
M. ravelobensis	Ravelobe	M73	-16.315202	46.822935	M	X			
M. ravelobensis	Ravelobe	M79	-16.312672	46.821591	M	X	X		
M. ravelobensis	Ravelobe	M81	-16.312081	46.821911	M	X	X		
M. ravelobensis	Ravelobe	M90	-16.31107	46.82303	M	X	X		
M. ravelobensis	Ravelobe	M95	-16.308149	46.805682	M	X	X		
M. ravelobensis	Ravelobe	F20	-16.300819	46.821725	F	X	X		
M. ravelobensis	Ravelobe	F21	-16.30211	46.819893	F	X	X		
M. ravelobensis	Ravelobe	F22	-16.301641	46.819766	F	X	X		
M. ravelobensis	Ravelobe	F25	-16.301265	46.81883	F	X	X		
M. ravelobensis	Ravelobe	F30	-16.302452	46.815375	F	X	X	X	
M. ravelobensis	Ravelobe	F41	-16.314803	46.822804	F	X	X	X	
M. ravelobensis	Ravelobe	F62	-16.30906	46.824098	F	X	X		
M. ravelobensis	Ravelobe	F77	-16.31355	46.8218	F	X	X		
M. ravelobensis	Ravelobe	F80	-16.311509	46.821839	F	X	X	X	
M. ravelobensis	Ravelobe	F82	-16.31022	46.821199	F	X	X		
M. ravelobensis	Ravelobe	F83	-16.308954	46.820228	F	X	X		X

M. ravelobensis	Ravelobe	F87	-16.307968	46.819708	F	X	X
M. ravelobensis	Ravelobe	F88	-16.311903	46.823296	F	X	X

Table S9. Information regarding the number of raw reads obtained from the Illumina sequencing (# raw reads), the number of reads that pass the quality filters (# reads after filtering), the number of reads that were maintained after the read alignment against the reference genome (# reads after filtering), and the final number of reads that were kept for downstream analyses (# reads without PCR duplicates) for the 22 unrelated *M. murinus* samples (7 Ravelobe and 15 Ankomakoma).

Study site	Sample ID	# raw reads	# reads after filtering	# reads after mapping	# reads without PCR duplicates
Ravelobe	M53	4706762	4176302	3959272	2959797
Ravelobe	M54	10173474	8439824	7803553	6675179
Ravelobe	M59	6221496	4931036	4662680	3649993
Ravelobe	M91	4131552	3683558	3561718	2752535
Ravelobe	M92	7657852	6839834	6574624	4718290
Ravelobe	M93	4034328	3593830	3473117	2678990
Ravelobe	F94	3529168	2963270	2714048	2323009
Ankomakoma	F104	6523524	5395046	4985213	4283736
Ankomakoma	F106	11899328	10357272	9763181	7355217
Ankomakoma	F107	15803430	13760734	12996027	9619186
Ankomakoma	F108	5012064	4386188	4133430	3332821
Ankomakoma	F118	4193164	3548674	3304485	2955094
Ankomakoma	F145	14674322	12804302	12062027	8950340
Ankomakoma	F148	8909372	7412438	6866175	5909654
Ankomakoma	M109	13931648	12186646	11515574	8578470
Ankomakoma	M110	4345738	3688712	3435409	3069805
Ankomakoma	M112	13315206	11530986	10864451	8104711
Ankomakoma	M115	4131774	3486058	3250098	2910721
Ankomakoma	M116	3600044	3534356	3187171	2862544
Ankomakoma	M143	14745302	12801252	12041723	8932595
Ankomakoma	M147	11613968	10105056	9517477	7209773
Ankomakoma	M153	7082780	6224776	5860194	4512990

Table S10. Information regarding the number of raw reads obtained from the Illumina sequencing (# raw reads), the number of reads that pass the quality filters (# reads after filtering), the number of reads that were maintained after the read alignment against the reference genome (# reads after filtering), and the final number of reads that were kept for downstream analyses (# reads without PCR duplicates) for the 56 unrelated *M. ravelobensis* samples (31 Ravelobe and 25 Ankomakoma).

Study site	Sample ID	# raw reads	# reads after filtering	# reads after mapping	# reads without PCR duplicates
Ravelobe	F20	28528772	7113482	6772011	4831626
Ravelobe	F21	21156854	4235984	4049956	3087415
Ravelobe	F22	5558098	4720766	4471175	3947638
Ravelobe	F25	58123672	8501128	8116708	5739055
Ravelobe	F30	8129904	7266498	6926482	4927203
Ravelobe	F41	8838044	7887444	7535708	5354344
Ravelobe	F62	5080140	4156016	3968820	3589260
Ravelobe	F77	5916048	4894924	4694210	2923671
Ravelobe	F80	4570176	4095462	3919926	2900030
Ravelobe	F82	9305280	7881754	7481544	6502397
Ravelobe	F83	7159666	6400234	6132212	4413963
Ravelobe	F87	3324066	2824308	2688945	2481757
Ravelobe	F88	4374028	3650898	3471998	3187091
Ravelobe	M18	8466752	7197946	6803186	5896938
Ravelobe	M19	71414688	8124844	7757127	5481242
Ravelobe	M29	5281620	4406536	4167438	3693149
Ravelobe	M42	4590576	3891020	3680040	3349695
Ravelobe	M48	5729448	4577840	4349856	3454207
Ravelobe	M52	5033296	4056096	3839551	3050626
Ravelobe	M55	6788952	6054820	5784430	4160609
Ravelobe	M56	5811554	5184500	4951429	3567787
Ravelobe	M57	8610870	7348548	6387468	5550607
Ravelobe	M58	8061824	6720560	6367259	5634880
Ravelobe	M60	6448470	5759526	5537053	4008336
Ravelobe	M67	7668306	6525312	6188608	5401009
Ravelobe	M68	6804362	6089804	5856734	4206378
Ravelobe	M73	4747160	3952624	3774305	3417907
Ravelobe	M79	6891944	5861178	5596801	4950520
Ravelobe	M81	8227234	6811972	6481569	5749194
Ravelobe	M90	3760890	3172790	3026282	2793325
Ravelobe	M95	8047384	6742558	6425237	5720089
Ankomakoma	F98	3631366	3102596	2958474	2729584
Ankomakoma	F99	6485154	5663834	5434859	4541666
Ankomakoma	F119	13961756	11407326	10889993	8392315
Ankomakoma	F126	11362500	9940250	9477176	7323835
Ankomakoma	F129	16606000	14596350	13905707	10379141
Ankomakoma	F132	14491200	12659586	12098730	9206957

Ankomakoma	F157	10015268	8563018	8150602	6580566
Ankomakoma	F165	12379618	10804546	10269556	7871948
Ankomakoma	F166	12478288	10948304	10416323	7951320
Ankomakoma	F169	12572186	11077374	10523370	8098503
Ankomakoma	M101	13423526	11783214	11238384	8587234
Ankomakoma	M102	12391962	10874142	10345348	7919043
Ankomakoma	M120	8788492	7357852	6977728	6167652
Ankomakoma	M121	12935332	11216794	10707218	8238080
Ankomakoma	M127	17003312	14836462	14131162	10487904
Ankomakoma	M130	7593208	6352198	6005767	5314352
Ankomakoma	M135	15682380	13802594	13136834	9819884
Ankomakoma	M138	16178616	14210212	13501541	10100636
Ankomakoma	M140	8369782	7419560	7040799	5478473
Ankomakoma	M141	15740714	13779016	13112996	9814927
Ankomakoma	M154	9530816	8278512	7887631	6407310
Ankomakoma	M158	11404824	10008818	9504542	7290555
Ankomakoma	M159	13226040	11641646	11062503	8412749
Ankomakoma	M161	15064372	13240982	12589968	9469711
Ankomakoma	M163	7814650	6792216	6482997	5305447

Supplementary references

1. Nei M. Estimation of average heterozygosity and genetic distance from a small number of individuals. Genetics. 1978;89:583–90.

2. Excoffier L, Lischer HEL. Arlequin suite ver 3.5: a new series of programs to perform population genetics analyses under Linux and Windows. Mol Ecol Resour. 2010;10:564–7.

3. Lischer HEL, Excoffier L. PGDSpider: an automated data conversion tool for connecting population genetics and genomics programs. Bioinformatics. 2012;28:298–9.

4. Fredsted T, Pertoldi C, Schierup MH, Kappeler PM. Microsatellite analyses reveal fine-scale genetic structure in grey mouse lemurs (*Microcebus murinus*). Mol Ecol. 2005;14:2363–72.

5. Olivieri GL, Sousa V, Chikhi L, Radespiel U. From genetic diversity and structure to conservation: Genetic signature of recent population declines in three mouse lemur species (*Microcebus spp.*). Biol Conserv. 2008;141:1257–71.

6. Radespiel U, Schulte J, Burke RJ, Lehman SM. Molecular edge effects in the Endangered golden-brown mouse lemur *Microcebus ravelobensis*. Oryx. 2019;53:716–26.

7. Luo Z, Zhou S, Yu W, Yu H, Yang J, Tian Y, et al. Impacts of climate change on the distribution of Sichuan snub-nosed monkeys (*Rhinopithecus roxellana*) in Shennongjia area, China. Am J Primatol. 2015;77:135–51.

8. Biesack EE. Population Structure of Lethrinus Lentjan (*Lethrinidae, Percoidei*) Across the South China Sea and the Philippines Is Detected With Lane- Affected RADSeq Data. 2017.

9. Bagley RK, Sousa VC, Niemiller ML, Linnen CR. History, geography and host use shape genomewide patterns of genetic variation in the redheaded pine sawfly (*Neodiprion lecontei*). Mol Ecol. 2017;26:1022–44.

10. Garg KM, Chattopadhyay B, Koane B, Sam K, Rheindt FE. Last Glacial Maximum led to community-wide population expansion in a montane songbird radiation in highland Papua New Guinea. BMC Evol Biol. 2020;20:82.

11. Aleixo-Pais I, Salmona J, Sgarlata GM, Rakotonanahary A, Sousa AP, Parreira B, et al. The genetic structure of a mouse lemur living in a fragmented habitat in Northern Madagascar. Conserv Genet. 2019;20:229–43. doi:10.1007/s10592-018-1126-z.

12. Evanno G, Regnaut S, Goudet J. Detecting the number of clusters of individuals using the software STRUCTURE: A simulation study. Mol Ecol. 2005;14:2611–20.

13. Google Earth [online]. Ankaranfantsika National Park, Madagascar. DigitalGlobe 2012. https://earth.google.com/web/. Accessed 26 Apr 2021.

14. Vieira FG, Fumagalli M, Albrechtsen A, Nielsen R. Estimating inbreeding coefficients from NGS data: Impact on genotype calling and allele frequency estimation. Genome Res. 2013;23:1852–61.
